# A high-density linkage map construction in guava (*Psidium guajava* L.) using genotyping by sequencing and identification of QTLs for leaf, peel, and pulp color in an intervarietal mapping population

**DOI:** 10.3389/fpls.2024.1335715

**Published:** 2024-02-27

**Authors:** Malarvizhi Mathiazhagan, Dayanandhi Elangovan, Vasugi Chinnaiyan, Kodthalu Seetharamaiah Shivashankara, Darisi Venkata Sudhakar Rao, Kundapura Venkataramana Ravishankar

**Affiliations:** ^1^ Division of Basic Sciences, ICAR-Indian Institute of Horticultural Research, Bengaluru, India; ^2^ Centre for Post-graduate Studies, Jain (Deemed-to-be) University, Bengaluru, India; ^3^ Division of Fruit Crops, ICAR-Indian Institute of Horticultural Research, Bengaluru, India; ^4^ Division of Post Harvest Technology and Agricultural Engineering, ICAR-Indian Institute of Horticultural Research, Bengaluru, India

**Keywords:** guava, genotyping by sequencing, SNP markers, linkage map, leaf color, peel color, pulp color, quantitative trait loci

## Abstract

*Psidium guajava* L. is an important fruit crop in the tropical and subtropical regions of the world. The advanced breeding methods are not employed for important commercial traits like peel and pulp color, seed hardiness, fruit size, etc., due to the scarcity of genome-wide molecular markers and high-density linkage maps. In this study, we employed single-nucleotide polymorphism (SNP) markers and identified quantitative trait loci (QTL) regions that are associated with color traits of leaf, peel, and pulp in the guava intervarietal mapping population. The mapping population was developed from the contrasting genotypes of fruit and leaf color. Variations in color among the segregating hybrids were recorded both visually and using a Color reader. A high-density linkage map of guava was constructed using the SNP markers from genotyping by sequencing (GBS) of 150 hybrid individuals of the cross ‘Arka Poorna’ (green) x ‘Purple Local’ (purple). The integrated linkage map consisted of 1426 SNPs mapped on 11 linkage groups (LG), spanning a total distance of around 730 cM with an average of 129.6 markers per LG. Through QTL analysis for color traits, a minor QTL region was identified for visually scored leaf color and peel color on LG1, whereas a major QTL was detected for pulp color in LG4. The Hunter color values (L* and, a*) also had major QTLs with overlapping marker intervals for leaf and peel colors, establishing the association of SNP markers to the trait. The QTLs harbored genes and transcription factors involved in lycopene and anthocyanin pigment biosynthesis. This is the first report of a high-density linkage map based on SNP markers in guava and QTL mapping for color characters in leaf, fruit peel and pulp. The genotyping information generated in this study can aid in genetic engineering and marker-assisted breeding in guava.

## 1 Introduction

Guava (*Psidium guajava* L.) is a perennial tree species grown across the tropical and subtropical regions of the world. It is one among the most produced fruit crops across the globe. Due to its hardy nature, it gets adapts well to different climatic and soil conditions. Guava fruits are nutritionally superior to many tropical fruits with high vitamin C, phenols, flavonoids, good antioxidant content, dietary fibers, and pharmacological properties (Rojas-Garbanzo et al., 2021; Mathiazhagan et al., 2023). Guava belongs to the family Myrtaceae that contains ~150 genera, of which the ‘Common Guava’ (*P. guajava* L.), ‘Cattley guava’ (*P. cattleianum* Sabine) ‘pear guava’ (*P. pyriferum* L.) and ‘Chinese guava or Costa Rican guava’ (*P. friedrichsthalianum* L.) are some of the important species. Heterozygosity of the *Psidium* species facilitated a wide variation in fruit traits among the germplasm across the world. The *P. guajava* L. has a diploid chromosome number of 2*n*=22 with a genome size of ~450 Mb (Coser et al., 2012; Feng et al., 2021).

In the case of guava, color of the fruit attracts and can be consumed as both fresh and processed products (Toppo et al., 2018). But guava is a climacteric crop, which is unsuitable for long-term storage and export as a fresh fruit (FAO, 2023). Guava is a heterozygous crop with cross pollination to the tune of 35%, which in turn has given rise to high variability in different fruit characters among the germplasm and wild species across the globe (Rajan et al., 2005; Ran et al., 2017). Hence, exploitation of available germplasm is essential for the development of hybrids that have attractive pulp color, peel color, fruit size, pulp content, reduced seed content and longer shelf life to sustain the demand from industry and export markets.

During ripening, fruits exhibit a noticeable color change because of rapid fluctuations in different pigment levels, due to the breakdown of chlorophyll and accumulation of carotenoids and anthocyanins (Choo, 2018). Carotenoids are terpenoid compounds synthesized through methylerythritol-phosphate (MEP) pathway. Anthocyanins are flavonoid compounds synthesized through phenylpropanoid pathway that renders red, purple or blue coloration to leaf, flowers and fruits (Albert et al., 2022). The pigment production in plants is under strong regulatory control of transcription factors (TFs) like myb, bHLH, WD repeats, NAC, etc (Zhao et al., 2022). The carotenoid and anthocyanin biosynthetic pathways and the genes associated are well studied in crops like tomato (Gonzali and Perata, 2021), apple (Ampomah-Dwamena et al., 2022), strawberry (Labadie et al., 2022) and peach (Zhao et al., 2020) for deciphering fruit skin and flesh color. However, the pigment biosynthetic pathways in leaf, peel and pulp color in guava remains unexplored and hence, requires detailed studies to understand the regulatory mechanisms.

Molecular markers like AFLP (Amplified Fragment Length Polymorphism), SSR (Simple Sequence Repeats), RAPD (Random Amplified Polymorphic DNA) and SRAP (Sequence-related Amplified Polymorphism) are used for the genetic diversity and molecular mapping in guava (Padmakar et al., 2015; Kherwar et al., 2018; and Kumar et al., 2020). It plays an important role in early screening of progenies to curtail a long and exhaustive conventional screening method. The first molecular linkage map of guava contained 167 AFLP markers in the integrated map and aided in the identification of QTLs for vegetative characters (Valdés-Infante et al., 2003). From then on, the linkage maps were constructed which helped in identifying QTLs related to tree and fruit quantitative traits (Rodriguez et al., 2007; Padmakar et al., 2016; and Maan et al., 2023). However, these studies have very low coverage and less number of markers on LGs. Interestingly, QTLs linked to the color variations in leaf, fruit peel and pulp remain unexplored as yet in guava except for a recent work on leaf anthocyanin reflective index by Sohi et al. (2022). Hence, the development of markers related to color traits will be a crucial step toward breeding of varieties with colored peel and pulp, which has more consumer preference and is suitable for making various processed products in short time.

Although the genome assembly of guava is available (Feng et al., 2021), the location of QTLs/genes associated to color traits is not yet clear. Hence, adapting next generation sequencing (NGS) based genotyping methods like genotyping by sequencing (GBS) and constructing high-density linkage maps becomes vital for precise identification of markers linked to the traits. GBS is a cost-effective strategy which provides sufficient genome coverage enabling identification of large number of SNPs throughout the genome (Elshire et al., 2011). Hence, SNP marker-based linkage maps in plants are highly dense, saturated, unambiguous, and informative (Srivastav et al., 2023).

In this study, we constructed a high-density linkage map of guava using GBS data generated for intervarietal segregating hybrids developed from the parents ‘Arka Poorna’ x ‘Purple Local’ with contrasting characters like leaf, peel, and pulp color and further identified QTL hotspots associated with color-related traits.

## 2 Materials and methods

### 2.1 Plant material

**Figure 1:**
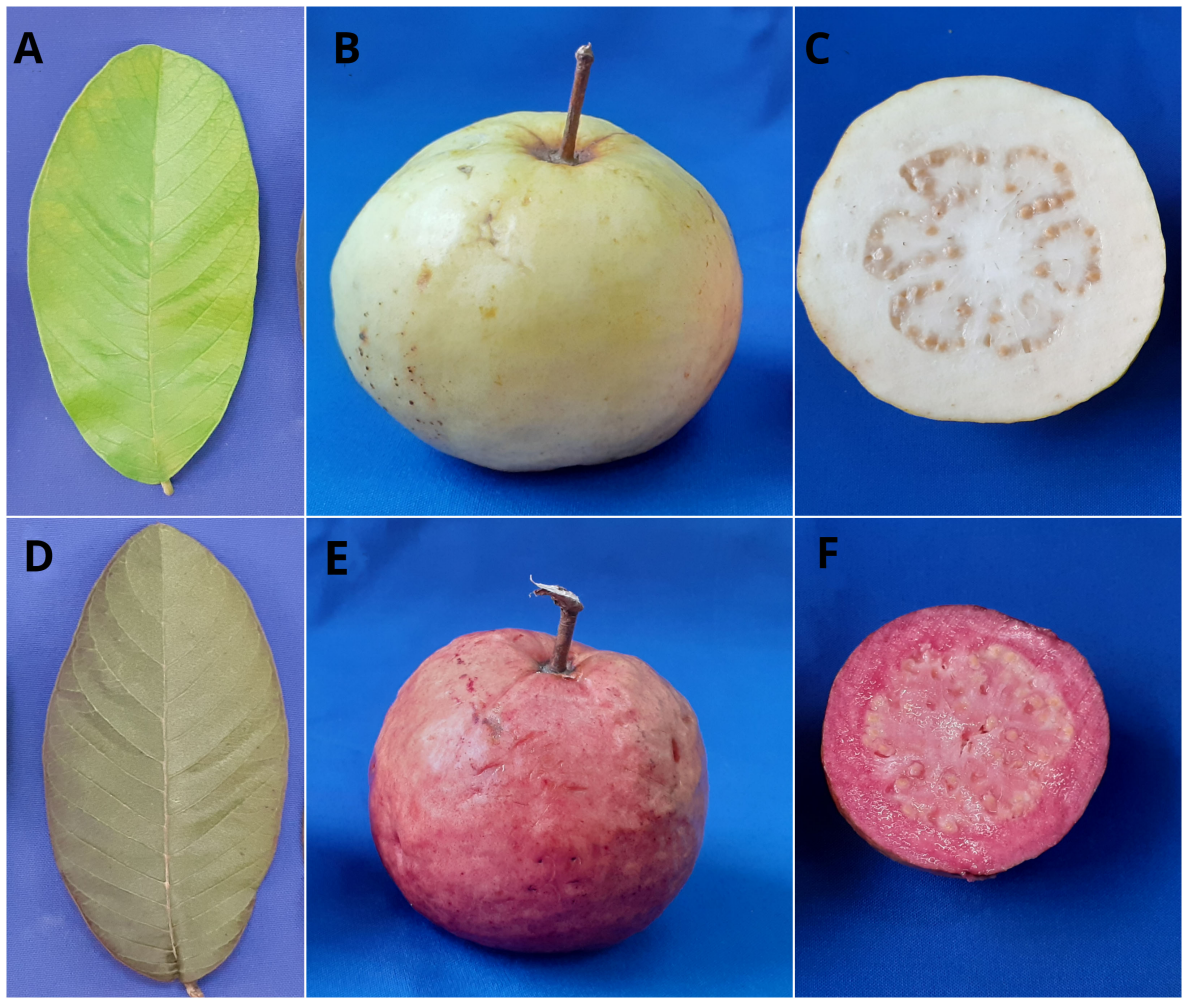
Variations in leaf, peel, and pulp color of guava parental genotypes. **(A-C)**- Female parent, Arka Poorna with green leaf, yellow peel, and white pulp; **(D-F)** – Male parent, Purple Local with grayed purple leaf, purple peel, and purple pulp).

Two contrasting guava cultivars, ‘Akra Poorna’ (white pulp) and ‘Purple Local’ (purple pulp) were crossed to generate 250 intervarietal progenies. The parents and the segregating hybrids were maintained at the fruit breeding block of ICAR-Indian Institute of Horticultural Research (IIHR), Bengaluru, India (13°8’3,984” N, 77°29’23.928” E). The segregating mapping population was transplanted in the field during the year 2020 in a row trial with a spacing of 2m x 1m. We have planted them as a row trial, as each individual is genetically different. The female parent ‘Akra Poorna’ is a cultivar from the progeny selection from the cross, ‘Purple Local x Allahabad Safeda’. The plant is characterized by green leaves and white-pulped fruits with yellow peel. The male parent ‘Purple Local’ is characterized by greyish purple-colored leaves, purple peel, and pulp with soft seeds. Both varieties are contrasting for leaf color, peel color and pulp color. Hence, they were chosen for the development of segregating progenies (**Figure 1**). Two-year-old randomly selected 150 intervarietal hybrids were used for GBS and QTL identification. Phenotypic data were collected for one season from 150 individuals during the months of September–November 2022. We have taken quantitative observations on leaf, peel, and pulp color in three replications. For visual scoring of leaf, peel, and pulp color, we employed 10 replications from each segregating individual.

### 2.2 Phenotyping for leaf and fruit color characters

The data on color was scored visually and also using a Color reader (NR 200 Color reader, 3NH Technologies, China) (**Table 1**). For visual scoring of the leaf color, observations were made on the 4^th^ mature leaf from the tip of 10 different branches. For the fruit peel color and pulp color, 10 fully ripened fruits from different branches throughout the whole fruiting season from each individual progeny and the parents were recorded. The color space values of leaf, peel and pulp were recorded in replicates of three using the Color reader. The Color reader measures color as L*, a*, and b* color space values. Value ‘L*’ denotes the brightness (0, black; 100, white), ‘a*’ denotes the red color (+ve) and the green color (–ve), and ‘b*’ denotes the yellow color (+ve) and blue color (–ve) (Nawaz et al., 2020). Correlation analysis was conducted to understand the correlation between visual color scores and different color space values (L*, a*, b*) from Color reader using metan package v.1.18.0 in R program (Olivoto and Lúcio, 2020). Descriptive statistics, frequency distribution, Shapiro–Wilk test of normality, analysis of variance, genotypic coefficient of variability, phenotypic coefficient of variability, heritability (broad sense) and genetic gain as percent of mean (GAM) for the color space values of the 150 individuals and parents were studied using Variability package v.0.1.0 in R program (Popat et al., 2020).

**Table 1:** Traits evaluated in the segregating mapping population.

Traits evaluated	Abbreviation	Variable type	Measurement description
Qualitative traits- visual scoring			
Leaf color	LC	Categorical	1: Green and 2: Greyed purple
Peel color	SC	Categorical	1: Yellow and 2: Purple
Pulp color	PC	Categorical	1: White 2: Purple and 3: Red
Quantitative traits-color space values			
Leaf- L*, a*, b*	LL, LA, LB	Continuous	L*: 0 to 100;
Peel- L*, a*, b*	PEL, PEA, PEB	Continuous	a*: +120 to -120;
Pulp- L*, a*, b*	PUL, PUA, PUB	Continuous	b*: +120 to -120

L*, luminosity (100, full brightness; 0, full darkness); a*, red (+), green (−) color; b*, yellow (+) and blue (−) color attribute.

### 2.3 GBS and variant calling

Total genomic DNA was isolated from young leaves of both parents ‘Arka Poorna’ and ‘Purple Local’, and 150 intervarietal segregating hybrids using a slightly modified CTAB method (Ravishankar et al., 2000). The isolated DNA samples were treated with RNAse (10 *μ*g/mL) and quantified using UV/Vis Nano spectrophotometer (Microdigital, Korea) at 260 nm. GBS libraries were prepared by restriction digestion of the DNA using *Ape*K1 enzyme. The library preparation and sequencing were carried out based on the manufacturer’s protocol (Clevergene Biocorp, Bengaluru, India) and sequenced on Illumina Novaseq 6000 sequencing platform based on paired end sequencing chemistry with a read length of 150 bp. The raw reads were processed with Fastp v0.20.1 (Chen et al., 2018) to remove low-quality bases and adapter sequences, followed by alignment against the reference genome of ‘New Age’ guava v.11.23 (Feng et al., 2021). Variant calling was performed using the STACKS ref_map v2.55 pipeline (Rochette et al., 2019). The identified SNPs were filtered further to remove low-quality SNPs and duplicates. The filtering criteria, namely minor allele frequency (MAF) at 0.05, maximum missing genotypes at 10%, and Hardy–Weinberg equilibrium (HWE) at 0.001 were employed. The genotype phasing was carried out using Beagle software v5.4 (Browning et al., 2018).

### 2.4 Linkage map construction

The linkage map construction was performed employing JoinMap v.5.0 software (Van Ooijen, 2011). The filtered SNPs were converted to cross-pollinator (CP) model of JoinMap using next generation sequencing eclipse plugin tool (NGSEP core v.3.3.2) (Tello et al., 2019). The segregation types included <lmxll>, where the locus is heterozygous in only first parent; <nnxnp>, where the locus is heterozygous in second parent; and <hkxhk>, where the locus is heterozygous in both parents, with two distinct alleles. An integrated linkage map was constructed with the logarithm of odds (LOD) set in the range 16–20. The genetic distances were estimated using the Kosambi mapping function and regression mapping for the ordering of markers in each LG.

### 2.5 QTL analysis

The QTL analysis for the phenotypic traits was performed using MAPQTL v6.0 software (Van Ooijen, 2009). The qualitative and quantitative traits were assessed based on the non-parametric Kruskal–Wallis test (*P* < 0.01). Further, the QTLs were confirmed using interval mapping. The genome-wide LOD threshold was set to identify the significant QTLs for each trait under study by employing 1000 permutation tests.

### 2.6 Identification and functional annotation of candidate genes

We have analyzed 100 Kb regions surrounding significant SNPs in the QTL regions. The sequences were retrieved from the reference genome (Feng et al, 2021) and BLAST searched in NCBI and UNIPROT databases to identify the candidate genes associated to the markers linked to the color traits. The functional annotation of genes was carried out through GO Ontology and KEGG pathway enrichment analysis using ShinyGO web server (Ge et al., 2020).

## 3 Results

### 3.1 Phenotyping for color characters of leaf, peel and pulp

**Figure 2:**
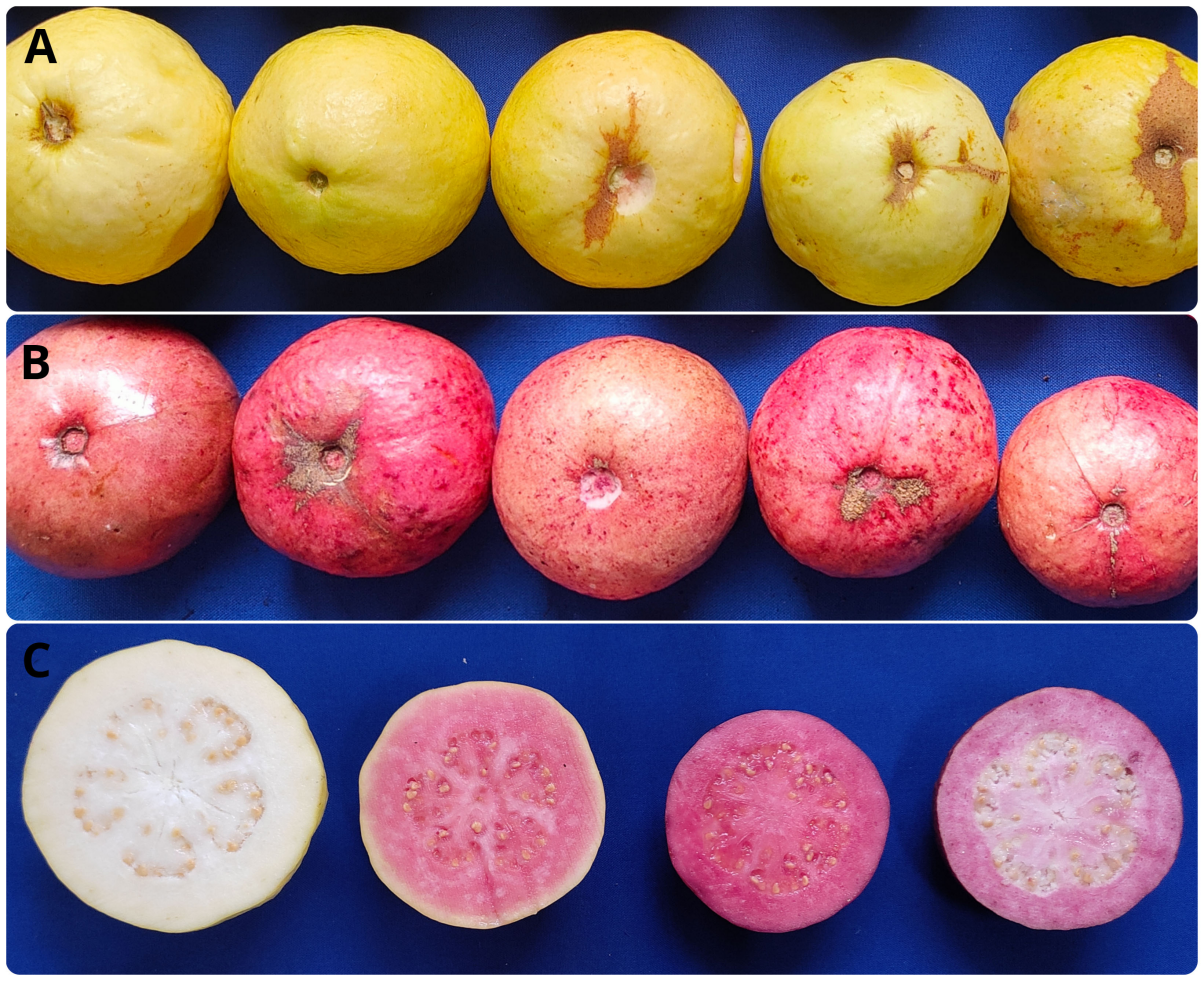
Variations in fruit peel and pulp color of intervarietal hybrids from the cross Arka Poorna x Purple Local. **(A)** Yellow peeled hybrids; **(B)** Purple peeled hybrids; **(C)** Hybrids with white, red, purple and light purple pulp.

**Figure 3:**
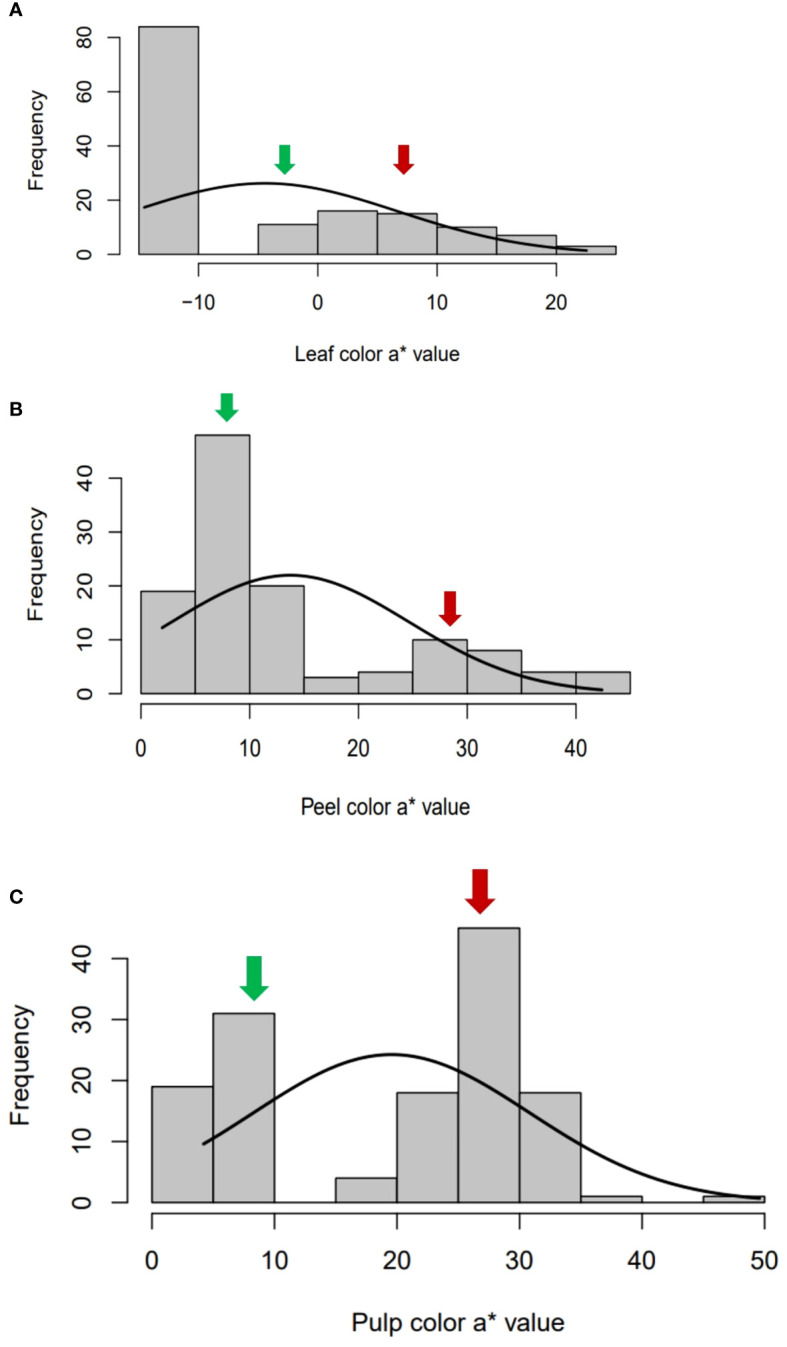
Bimodal frequency distribution of color space a* values in **(A)** leaf, **(B)** peel and **(C)** pulp in the intervarietal hybrids of the cross AP x PL. Green arrow- values of female parent, Arka Poorna; red arrow: values of male parent, Purple local.

To study the parameters, from both qualitative and quantitative perspectives, phenotyping of leaf, peel and pulp color by using visual scoring and color space values was adopted. Three qualitative traits, namely leaf color (LC), peel color (SC) and pulp color (PC) were scored visually. The green-leaf plants produced only yellow peeled fruits with white or red pulp, whereas the greyed-purple leaf plants comprised of only purple peeled fruits with purple pulp (**Figure 2**). Gradation in peel, pulp and leaf color was observed in the intervarietal progenies based on their color space values. The frequency distribution and the QQ plots of a* values of leaf, peel and pulp showed a bimodal distribution with two distinct distribution classes for the traits (green/purple leaf; yellow/purple peel; and white/colored pulp) (**Figure 3**, **Supplementary Figures 1**-**3**). The Shapiro–Wilk test also indicated that the observed color space values for leaf, peel and pulp follow a non-normal/skewed distribution (W= 0.80 to 0.92, *P ≤* 0.001). A moderate positive skewness was observed for color space traits, leaf a* value (LA), leaf b* value (LB), peel a* value (PEA), pulp L* value (PUL) and pulp b* value PUB, whereas the values were negatively skewed for leaf L* value (LL), peel L* value (PEL), peel b* value (PEB) and pulp b* value (PUA). Parents, ‘Arka Poorna’ and ‘Purple Local’ had different values for the color L* and a* in leaf, peel and pulp. Color a* value of leaf (LA), peel (PEA) and pulp (PUA) showed the highest coefficient of variation (CV%) among the hybrids with 106.18%, 79.40% and 57.45%, respectively (**Table 2**).

**Table 2:** Descriptive statistics of color space values of parents and intervarietal hybrids.

Color values	Arka Poorna♀	Purple Local♂	Intervarietal hybrid population (*n*=150)												
	Mean	Mean	Min.	Max.	Median	Mean	Skewness	Kurtosis	SD	SW (W*)	CV (%)	GCV (%)^a^	PCV (%)^a^	h^2^ (%)^b^	GAM (%)^c^
Leaf color space values															
L	38.34 ± 0.57	23.03 ± 0.35	20.29	41.49	33.2	31.44	-0.36	-1.21	5.46	0.92	17.35	16.10	17.74	82.76	29.60
a^#^	-1.42 ± 0.64	8.74 ± 6.18	-14.56	22.54	-12.1	-4.55	0.79	-0.82	11.10	0.80	106.18	60.49	84.35	51.44	89.38
b	29.56 ± 0.41	21.98 ± 0.56	11.39	88.71	32.0	32.15	0.94	2.19	12.37	0.92	38.47	35.77	38.98	84.21	67.62
Peel color space values															
L	71.97 ± 1.39	41.18 ± 0.89	20.44	74.24	66.4	58.24	-1.00	-0.50	15.41	0.80	26.45	22.67	24.59	84.98	46.31
a	9.04 ± 0.65	29.64 ± 4.15	1.94	42.41	9.39	13.64	1.20	0.16	10.83	0.81	79.40	69.13	77.63	79.31	129.20
b	37.99 ± 3.17	41.53 ± 1.11	18.62	63.21	41.4	41.22	-0.17	0.13	7.99	0.57	19.39	13.14	20.75	40.09	16.00
Pulp color space values															
L	69.34± 1.24	44.36 ± 2.03	38.29	78.56	54.5	58.57	0.26	-1.46	12.05	0.90	20.57	19.30	20.70	86.48	36.40
a	8.25 ± 0.88	26.09 ± 1.14	4.21	49.58	24.1	19.59	-0.22	-1.35	11.25	0.84	57.45	53.56	58.90	82.64	97.00
b	21.71 ± 2.40	14.65 ± 1.37	7.00	43.66	17.4	16.82	0.79	7.08	4.67	0.88	27.73	20.49	35.66	33.04	18.88

^#^Leaf a* recorded negative values for green leaves, hence data scaling was applied to calculate variability, heritability and genetic gain.

*, Shapiro–Wilk test statistic values at P ≤ 0.001 for all parameters except for peel b* values (P= 0.969).

a Variability ^<^10% (low), 10–20% (moderate), >20% (high).

b Heritability 0-30% (low), 31-60% (moderate), >60% (high).

c GAM ^<^10% (low), 10–20% (moderate), >20% (high).

SW, Shapiro–Wilk test of normality.

GCV, genotypic coefficient of variability; PCV, phenotypic coefficient of variability; h^2^, heritability in broad sense; GAM, genetic advance as a per cent of mean.

The analysis of variance pertaining to the quantitative color values of leaf, peel and pulp has indicated that the traits (L*, a*, b*) significantly vary among the genotypes (**Supplementary Table 1**). For variability estimates, all color attributes showed a higher phenotypic (PCV%) and genotypic coefficient of variability (GCV%) except for L* values of leaf and pulp which were found to be moderate. Similarly, the heritability (h^2^%) remained high among the L* and a* values of all the traits, whereas b* values presented moderate to low heritability. The genetic advance as percentage mean (GAM%) was observed to be high for the a*values of the peel (129.20%) and pulp (97%) while b* value of peel (16%) had the least GAM% (**Supplementary Figure 4**).

**Figure 4:**
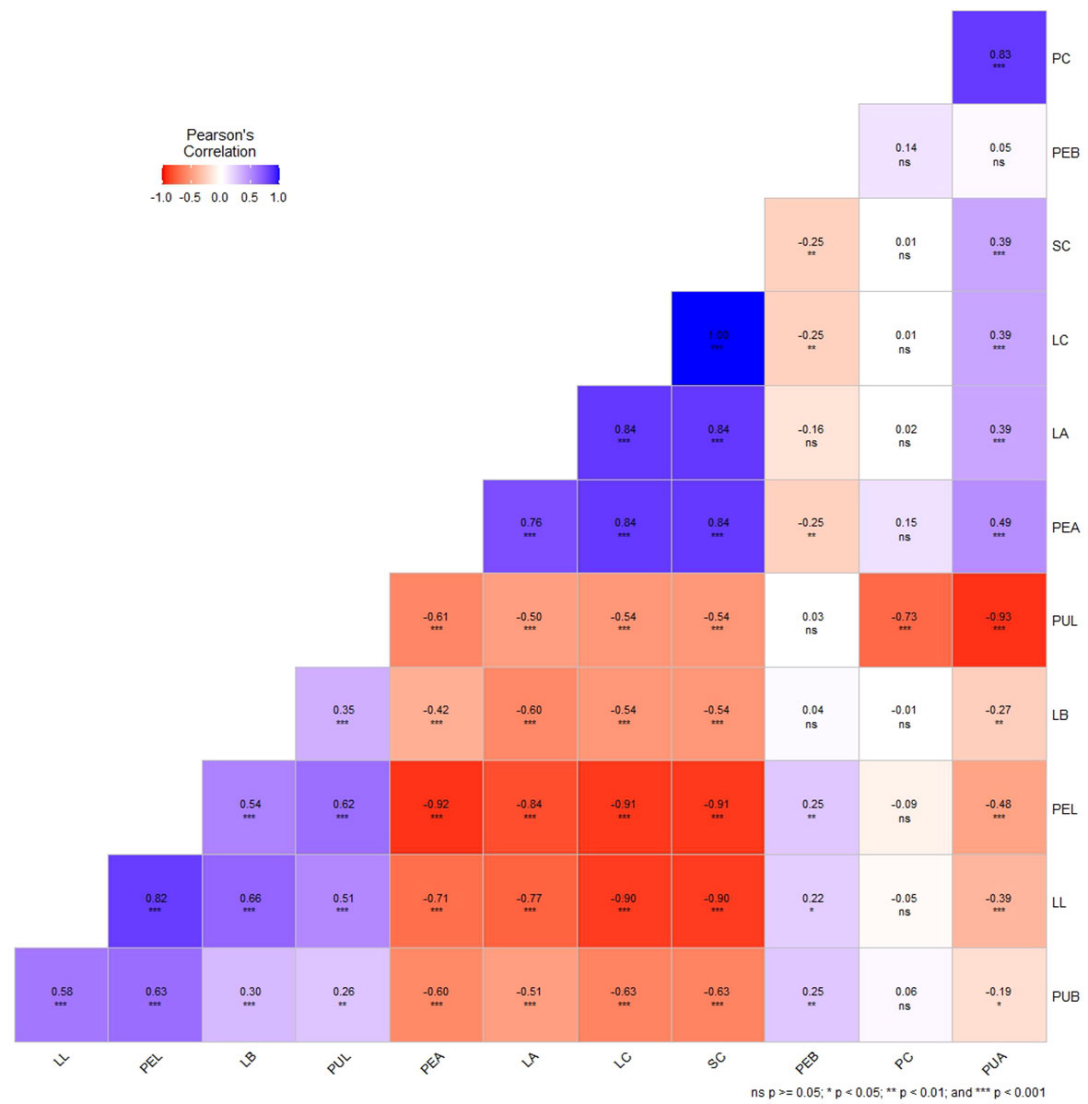
Correlation plot of Pearson’s correlation coefficient of qualitative (visual scores) and quantitative traits (color space values - L*, a*, b*) of leaf, peel, and pulp color in guava intervarietal hybrids; LC-Leaf color; SC- Peel color; PC- Pulp color; LL- Leaf L*value; LA- Leaf a* value; LB- Leaf b* value; PEL- Peel L* value; PEA- Peel a* value; PEB- Peel b* value; PUL- Pulp L* value; PUA- Pulp a* value and PUB- Pulp b* value. * P<0.05, ** P<0.01, *** P<0.001, ns- non-significant.

The correlation analysis between the visual scores and color space values of leaf, peel and pulp showed highly significant positive correlation values (*P*< 0.001) between the parameters, namely visually scored leaf color (LC) and peel color (SC) (r^2^ = 1.00, *P*< 0.001); LC, SC and PC with their corresponding a* values, LA (r^2^ = 0.81, *P*< 0.001), PEA (r^2^ = 0.84, *P*< 0.001) and PUA (r^2^ = 0.83, *P*< 0.001). In addition, significant negative correlations were recorded by color space value L* with the a* values of leaf (r^2^= –0.77), peel (r^2^= –0.92, *P*< 0.001) and pulp (r^2^= –0.74, *P*< 0.001); and, with LC (r^2^= –0.91, *P*< 0.001), SC (r^2^= –0.91, *P*< 0.001) and PC (r^2^= –0.93, *P*< 0.001) respectively (**Figure 4**). The b* values (LB, PEB and PUB) did not show any significant correlation with any of the parameters.

### 3.2 Genotyping by GBS and quality assessment

GBS of parents and the 150 hybrids generated raw reads with an average GC content of 50% (**Supplementary Table 2**). The average number of clean reads among the segregating hybrids was 7,863,663 with an average mapping per cent to the reference genome at 87.25%. Subsequently, the variant calling using the STACKS pipeline identified 1,19,343 SNPs. Filtration for MAF, missing values and HWE resulted in 34,525 good quality SNPs (16702 lm x ll, 14163 nn x np and 3660 hkxhk) for linkage map development (**Table 3**). The variant data generated in this study have been deposited in the European Variation Archive (EVA) at EMBL-EBI under accession number PRJEB70471.

**Table 3:** Details of segregating alleles mapped to reference genome.

Segregation code	SNP count	Percentage* (%)
lmxll	16702	48.37
nnxnp	14163	41.02
hkxhk	3660	10.604
Total	34525	

*Percentage of segregation code for total mapped loci.

### 3.3 Construction of high-density linkage map

**Figure 5:**
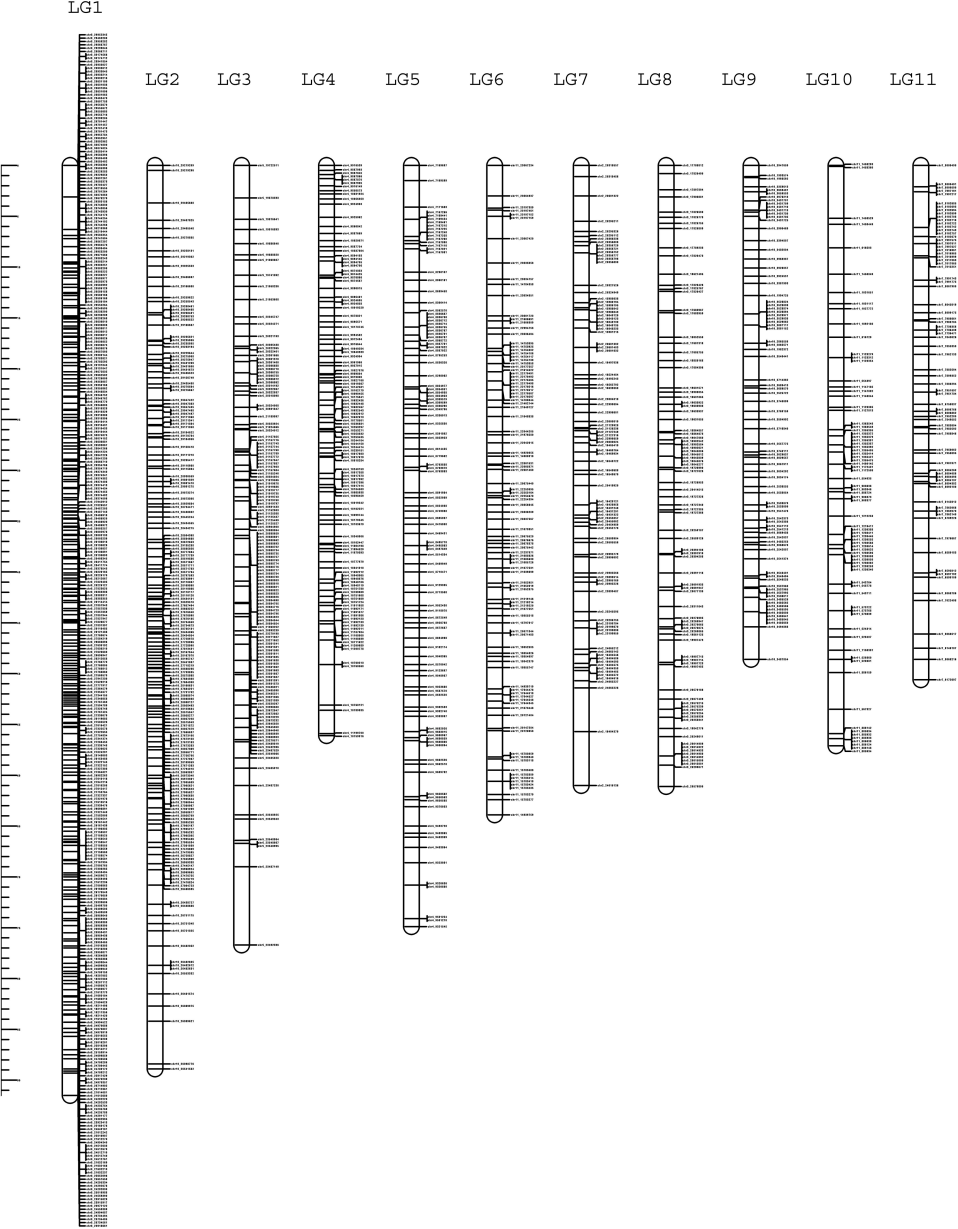
High-density linkage map of guava containing 11 LGs constructed using 150 individuals of intervarietal cross, ‘Arka Poorna’ x ‘Purple local’.

The high-density linkage map was constructed using JoinMap 5.0 software under CP population mode by employing 34,525 high quality SNPs after filtration. A total of 1426 SNPs were mapped to 11 LGs spanning a total length of 730.2 cM with LG1 being the largest and LG9 being the smallest (**Figure 5**; **Table 4**). The average length of the LGs was 66.3 cM. The marker interval ranged from 1.3 to 3.9 cM with an average marker interval of 1.8 cM between the markers. The number of markers per LG varied from 76 in LG11 to 358 in LG1 with the average of 129.6 markers in each LGs. The logarithm of odds (LOD) was set between 16 and 20 to order the markers in the LGs.

**Table 4:** Distribution of mapped markers in 11 LGs of guava.

LGs	Number of SNP markers	Total length (cM)	Average marker interval (cM)
LG1	358	91.5	3.9
LG2	185	88.8	2.0
LG3	141	76.7	1.8
LG4	120	56.1	2.1
LG5	108	74.9	1.4
LG6	102	63.9	1.5
LG7	90	61.0	1.4
LG8	87	61.1	1.4
LG9	82	48.5	1.6
LG10	77	57.1	1.3
LG11	76	50.6	1.5
Total	1426	730.2	–
Average	129.6	66.3	1.8

### 3.4 Identification of QTLs for leaf, peel and pulp color

**Figure 6:**
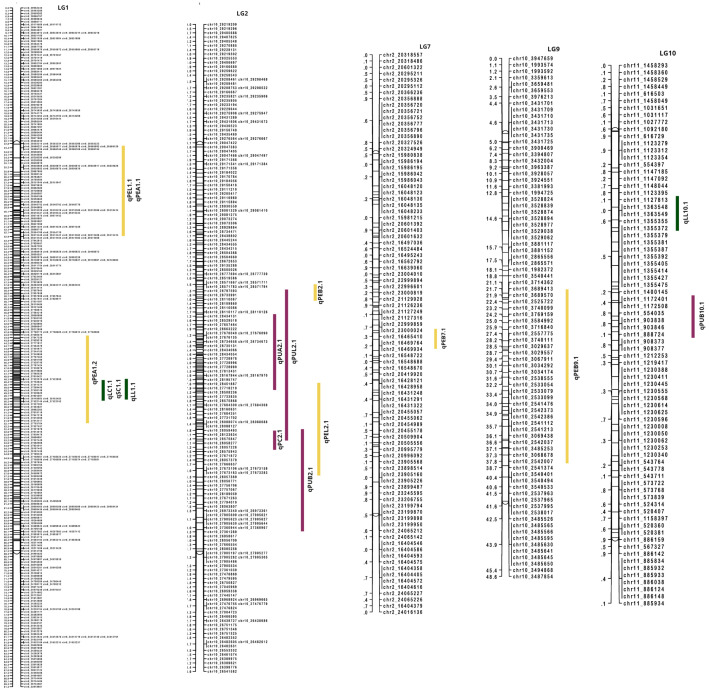
QTLs identified for color traits in guava. Leaf color traits are represented in green bars; peel color traits in yellow bars and pulp color traits in purple bars.

By integrating the visually scored color parameters and Color reader color space values (L*, a*, b*) with the linkage map, 16 QTLs were identified across five LGs. The QTLs identified are summarized in **Table 5**. LG1 harbored a QTL at position 49.66 cM for both leaf color (qLC1.1) and peel color (qSC1.1) which contributed to 10.2% phenotypic variation for the trait. For pulp color, LG2 presented a QTL (qPC2.1) at position 52.05 cM that explained a phenotypic variation of 43.2% (**Figure 6**, **Table 5**). The observed phenotypic variations indicated that all the three QTLs identified for visually scored color traits were major QTLs (**Supplementary Figure 5**).

**Table 5:** QTLs identified for qualitative and quantitative color traits using interval mapping method.

Traits	QTL^a^	LG	Marker close to peak	Marker position (cM)	Marker interval	Marker LOD ^b^	PVE^c^ (%)	Kruskal–Wallis analysis	
								K*	Signif.^d^
Visual color scores									
Leaf color	qLC1.1	1	Chr8_27824291	49.66	Chr8_27754834- Chr8_28148003	3.53	10.2	5.497	**
Peel color	qSC1.1^#^	1	Chr8_27824291	49.66	Chr8_27753764- Chr8_28148003	3.53	10.2	5.497	**
Pulp color	qPC2.1	2	Chr10_28058277	52.056	Chr10_28058493- Chr10_28057228	18.41	43.2	61.076	*******
Leaf color space values									
L*	qLL1.1^#^	1	chr8_27824291	49.66	chr8_27754834- chr8_28148003	3.94	11.4	7.2	***
	qLL10.1	10	chr11_1363548, chr11_1363549	26.032	chr11_1127813- chr11_1355372	3.22	9.7	4.8	**
Peel color space values									
L*	qPEL1.1	1	chr8_28586129	23.412	chr8_28586227- chr8_29374340	4.03	14.3	4.48	**
	qPEL2.1	2	chr10_28575868	48.838	chr10_28451887- chr10_28056771	3.1	11.2	1.8	–
a*	qPEA1.1	1	chr8_28586129	23.412	chr8_28586227- chr8_29374340	4.45	15.7	7.1	***
	qPEA1.2^#^	1	chr8_27824291	49.66	Chr8_28212658- Chr8_27753764	3.11	11.2	5.2	**
b*	qPEB2.1	2	chr10_28797593	41.251	chr10_28571783- chr10_28110087	32.35	71.1	14.6	******
	qPEB9.1	9	chr10_3716840	25.925	chr10_3689413- chr10_2542007	3.74	12.6	1.6	–
	qPEB7.1	7	chr2_16469764	28.195	chr2_16469934- chr2_23000024	3.48	12.5	0.107	–
Pulp color space values									
L*	qPUL2.1	2	chr10_28434131	43.228	chr10_28797593- chr10_28058277	9.74	27.9	34.7	*******
a*	qPUA2.1	2	chr10_28434066	45.35	chr10_28434131- chr10_28508236	13.2	35.4	49.7	*******
b*	qPUB10.1	10	chr11_903846	31.76	chr11_1172401- chr11_888724	8.51	24	5.0	**
	qPUB2.1	2	chr10_27905644	59.114	chr10_28123634- chr10_26958617	5.62	17.2	7.8	***

L*, a*, b*- Color space values.

K*- Kruskal Wallis test statistic.

a QTLs named with ‘q’ as prefix followed by abbreviation of traits and LG number.

b Logarithm of odds (above the genome wide threshold LOD for each trait).

c Percentage of phenotypic variance explained by the QTL.

d significance levels: ** P<0.001; *** P<0.0005; **** P<0.0001, ***** P<0.00005, ****** P<0.00001, ******* P<0.000005.

#QTL detected for both visual color and color space values with overlapping marker intervals.

-Dash denotes no significance.

No significant QTLs for leaf a* and b* values were identified.

For color space values, QTLs were found across LG1, LG2, LG7, LG9 and LG10. In the leaf, only L* color value contained two QTLs (qLL1.1 and qLL10.1) in LG1 and in LG10 with the explained phenotypic variation at 11.4 and 9.7%, respectively. The QTL, qLChL1.1 in LG1 was present at the same position (49.66 cM) as in the visually scored leaf color QTL. In peel, all the color attributes contained major QTLs with the b value presenting a QTL (qPEB2.1) with the highest phenotypic variation of 71.1%. The PEL and PEA values of peel in LG1 shared an overlapping QTL region at position 23.412 cM. Similarly, in pulp, PUA value contained an overlapping QTL region (qPUA2.1) in LG2 (22 cM to 56 cM) with PUL value (qPUL2.1) and the visual pulp score QTL (qPC2.1). There are overlapping QTL clusters in LG1 and LG2 for the color traits that are detected both in visual scores and color space values.

### 3.5 Identification and functional annotation of candidate genes

**Figure 7:**
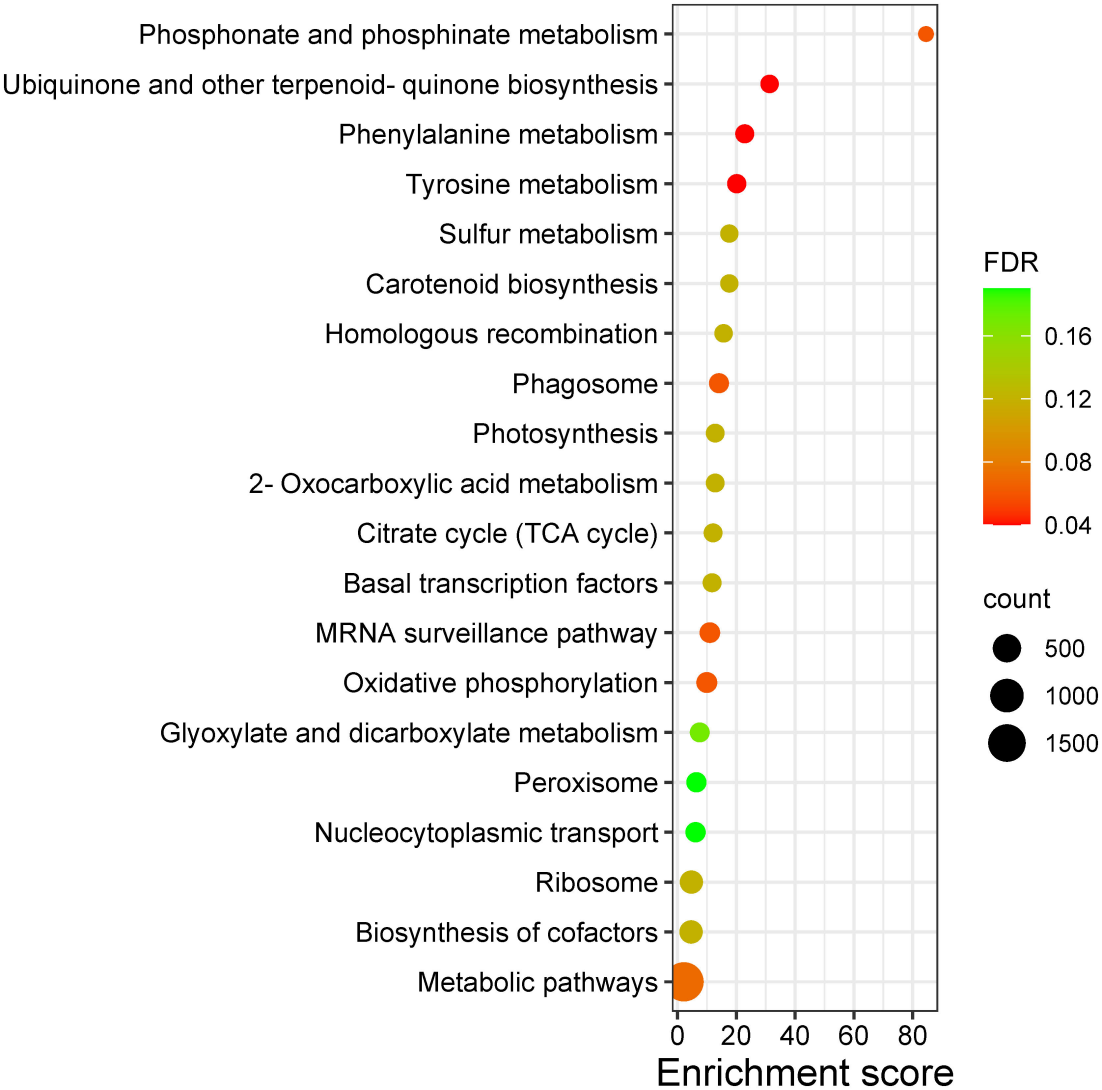
Bubble plot representing the KEGG pathways enriched in the QTLs regions of leaf, peel and pulp color traits in guava. ‘count’ indicates number of genes enriched in the pathway; ‘FDR’ indicates the False Discovery Rate.

The markers associated with leaf color trait QTLs were mapped to chromosomes 8 and 11 of reference genome, whereas the SNP markers linked to peel color were from chromosomes 2, 8 and 10. Pulp color SNPs were found mapped to chromosomes 10 and 11. Functional annotation of genes in the QTL regions of leaf, peel and pulp color identified several candidate genes and transcription factors involved in pigment formation. The leaf QTLs harbored genes like HD-ZIP, bZIP and ABC trasporter domain containing protein (**Table 6**). The peel QTL regions harbored TFs like bHLH, WD repeats, Myb and HD-ZIP, and structural genes like Phytoene synthase, 4-hydroxyphenylpyruvate dioxygenase (HPPD), pentacotripeptide-repeat region and glycosyltransferases. The pulp QTL regions also contained genes like HPPD, glucan endo-1,3-beta-D-glucosidase, Myb, AP2/ERFs, HD-ZIP, cytochrome p450 and NAC domain-containing protein. The GO ontology and KEGG pathway analysis revealed the enrichment of pathways like Phosphonate and phosphinate metabolism, tyrosine metabolism, Phenylalanine metabolism, Carotenoid biosynthesis, 2-Oxocarboxylic acid metabolism etc (**Figure 7**). In biological processes, carotenoid biosynthetic process, tetraterpenoid metabolic process and isoprenoid biosynthetic process related to pigment production were enriched. Molecular functions category was enriched with 4-hydroxyphenylpyruvate dioxygenase activity, geranylgeranyl-diphosphate geranylgeranyltransferase activity, citrate synthase activity, sulfur dioxygenase activity, UDP-glycosyltransferase activity etc (**Supplementary Tables 3**, **4**; **Supplementary Figures 6**–**8**).

**Table 6:** Candidate genes identified in the QTL regions for leaf, peel and pulp color in guava related to lycopene and anthocyanin biosynthetic pathways.

Trait	QTL name	Chromosome no.	Description	Functions	E value
Leaf color	qLC1.1 qLL1.1	8	Homeobox-leucine zipper protein MERISTEM L1-like	Regulation of flavonoid biosynthesis	9E-47
Leaf L*	qLL10.1	11	BZIP domain-containing protein	Regulation of Chlorophyll degradation	0
Peel color	qSC1.1 qPEL1.1 qPEA1.1	8	Homeobox-leucine zipper protein MERISTEM L1-like	Regulation of flavonoid biosynthesis	7E-50
Peel L*	qPEL2.1	10	Phytoene synthase	Key enzyme in Carotenoid biosynthesis	2E-157
			HTH Myb-type domain-containing protein	Regulation of flavonoid and carotenoid biosynthesis	8E-117
			NAC domain-containing protein	Regulation of anthocyanin and carotenoid accumulation	0
			4-hydroxyphenylpyruvate dioxygenase	Key enzyme in Shikimate pathway	0
Peel a*	qPEA1.2	8	Homeobox-leucine zipper protein MERISTEM L1-like	Regulation of flavonoid biosynthesis	
Peel b*	qPEB2.1	10	bHLH domain-containing protein	Regulation of flavonoid biosynthesis	0
			NB-ARC domain-containing protein	Regulation of signaling pathways, flavonoid and carotenoid biosynthesis	0.7
			WD repeat-containing protein 26 homolog	Regulation of flavonoid biosynthesis	0
			Homeobox-leucine zipper protein MERISTEM L1-like	Regulation of flavonoid biosynthesis	9.00E-45
Peel b*	qPEB9.1	10	S-acyltransferase, 2.3.1.225, Palmitoyltransferase	Regulation of protein localization, stability, and activity	0
			Glycosyltransferase	Plant growth regulation and development	3E-22
Peel b*	qPEB7.1	2	Pentatricopeptide-repeat region of PRORP domain-containing protein	Fruit ripening and flesh color development	0
Pulp color	qPC2.1	10	AP2/ERF domain-containing protein	Regulation of Chlorophyll degradation	4.00E-23
Pulp L*	qPUL2.1	10	Myb-like domain-containing protein	Regulation of flavonoid and carotenoid biosynthesis	0.002
			4-hydroxyphenylpyruvate dioxygenase	Key enzyme in Shikimate pathway	0
			NAC domain-containing protein	Regulation of anthocyanin and carotenoid accumulation	0
Pulp a*	qPUA2.1	10	NAC domain-containing protein	Regulation of anthocyanin and carotenoid accumulation	0
			HTH Myb-type domain-containing protein	Regulation of flavonoid and carotenoid biosynthesis	4E-109
			Glucan endo-1,3-beta-D-glucosidase	Fruit ripening	1E-91
Pulp b*	qPUB10.1	11	Zinc finger protein STAMENLESS 1-like	Regulation of flavonoid biosynthesis	0
			BZIP domain-containing protein	Regulation of Chlorophyll degradation	0
			Cytochrome P450	Regulation of flavonoid biosynthesis	0
Pulp b*	qPUB2.1	10	Homeobox-leucine zipper protein MERISTEM L1-like	Regulation of flavonoid biosynthesis	0
			AP2/ERF domain-containing protein	Regulation of Chlorophyll degradation	0

## 4 Discussion

### 4.1 Phenotyping of color traits in intervarietal population

Fruit color has great breeding and commercial importance in fruit trees like apple, mango, peach, plum, guava, citrus, pitaya, etc (Mathiazhagan et al., 2021). Breeding varieties for colored peel and pulp becomes quintessential for sustaining the demand and market share of guava. In apple, the availability of reference genomes, genetic linkage maps and association studies facilitated the identification of QTLs associated with fruit color (Chen et al., 2021). Similarly, in mango, various omics-based approaches (Mathiazhagan et al., 2022) and QTL mapping identified genes linked to fruit color (Srivastav et al., 2023). In our study, we crossed contrasting parents, ‘Arka Poorna’ and ‘Purple Local’ and developed a diverse mapping population that has highly variable leaf color (green and greyed purple), peel color (yellow and purple) and pulp color (white, purple and red). The observed variability in this segregating population made it highly suitable for genetic linkage map construction and QTL identification of color-related characters.

Phenotypic observations of hybrids demonstrated that leaf color and peel color were tightly linked, as purple-leaved plants produced only purple peeled fruits. Hence, color of the leaf can serve as morphological marker for determining the fruit peel color in the seedling stage in guava hybrids. In the case of pulp color, hybrids with purple peel exhibited only purple pulp but in varying degrees resembling the male parent ‘Purple Local,’ whereas yellow peeled hybrids presented fruits with white or red pulp suggesting that pulp color is controlled by multiple genes and has a polygenic inheritance in guava. Further, it can also be stipulated that colored pulp (red/purple) is dominant over white pulp in guava for the cross used in this study. These results contradicted Subramanyam and Iyer (1992), who observed the pulp color to be a monogenically inherited trait, but our results are in agreement with the observations of Thakre et al. (2023), which emphasized the polygenic nature of guava pulp color.

The visual scoring of leaf and fruit colors is the easiest and the most common practice when it comes to the evaluation of mapping populations, considering the size and complexity of trait variations (Ntladi et al., 2018 and Luo et al., 2020). Even though color is a qualitative trait, subjective scoring may not depict color variations in a population precisely. Studies have reported employing the Color reader for measuring color attributes like L*, a*, and b* for scoring and identification of QTLs for fruit peel color and pulp color in segregating mapping populations (Oh et al., 2023; Srivastav et al., 2023 and Zhang et al., 2019). In the present study, leaf and fruit colors were scored using both methods in order to identify the QTL regions with more accuracy. The distribution of color a* value for leaf, peel and pulp was bimodal since we used a population that bore two distinct phenotypes for these traits. Similar, bimodal distribution for fruit color traits was observed in segregating populations of apple (Kunihisa et al., 2019) and grapes (Underhill et al., 2020). The bimodal distribution of traits indicated that a major QTL region might be involved in the variations observed for the traits. The phenotypic coefficient of correlation between color space values and visual scores of peel and pulp indicated the direct influence of respective color a* values over these traits. High CV, GCV and PCV were observed for a* values of leaf, peel and pulp suggesting a higher variability for leaf, peel and pulp color among the segregating hybrids (**Table 2**). High heritability (h^2^) and GAM of the color traits, especially a* values showed that they can serve as a better indicator for the selection of progenies for future breeding strategies.

### 4.2 Linkage map construction using SNP markers

GBS has become a cost-effective approach for genotyping large numbers of samples, especially the mapping populations that are required for constructing linkage maps. GBS has been employed for constructing genetic linkage maps in several fruit tree species including apple (Oh et al., 2023), Japanese plum (Battistoni et al., 2022), Korean pears (Han et al., 2019), banana (Sampangi-Ramaiah et al., 2023) and in the guava’s closest related species eucalyptus (Zhu et al., 2023). In our study, GBS has been applied for the first time in SNP calling in guava mapping populations which enabled us to identify 119,343 SNPs in both parents and hybrids. In earlier reports, linkage maps of guava were constructed using AFLP markers (Valdés-Infante et al., 2003; Rodriguez et al., 2005). Later, the linkage maps were improved by using SSR and SRAP markers for constructing integrated and parental linkage maps (Ritter et al., 2010 and Padmakar et al., 2016).

In all the previous studies, the major drawback was that the linkage maps were constructed using conventional molecular markers like SSRs, AFLPs, SRAPs and InDels based primers that gave very low coverage of the map (Maan et al., 2023). The low coverage due to a smaller number of markers on the map limited the capability of associating a particular trait to the closest marker. Recently, Medeiros et al. (2022) constructed a genetic linkage map incorporating 203 SNPs generated from a DNA marker chip which also did not provide complete coverage of LGs. The GBS approach employed in our study has resulted in good genome coverage and genome-wide SNP identification when compared with the previous reports.

Stringent filtering enabled omission of low-quality SNPs and distorted markers from the linkage map. Even though GBS does not require a reference genome for mapping, however, its availability (Feng et al., 2021) has facilitated the proper alignment and ordering of SNPs in guava. The map has markers that are placed at short distances with the highest density of 3.9 markers/cM in LG1 and the lowest density in LG10 with 1.3 markers/cM. When compared to other published maps of guava that had an average marker distance between 3.2 and 29.6 (Padmakar et al., 2016 and Sohi et al., 2022), we were able to construct the linkage map with the average marker interval at 1.8 cM. Further, the map has been constructed with a LOD value between 16 and 20 and incorporated 1426 SNPs which is also high compared to the previously reported linkage maps (**Table 4**). The higher the LOD, the better the association of the markers to the traits. Therefore, this makes the present map one of the saturated and dense genetic linkage maps of guava as of date.

### 4.3 QTLs identification for leaf, peel and pulp color of fruit

For the visual color scores, two major QTLs were identified, one in LG1 for LC and SC, and other in LG2 for PC. Color space values of leaf and peel have detected the same QTL region in LG1 (marker, Chr8_27824291 at position 49.66 cM) as in LC and SC, indicating that this might be a potentially major effect QTL region controlling leaf and peel color in guava. These results along with phenotypic observations strongly suggested that both leaf and peel color are tightly linked traits. In addition, color space values have QTLs for leaf color in LG10 and for peel color in LG2, LG9 and LG7 indicating the polygenic control of the traits. Based on the nomenclature of the markers linked to the leaf and peel color trait, it can be inferred that the QTL regions for leaf and peel color may be present in the chromosomes 2, 8, 10 and 11 of guava genome (**Table 5**). Similar results were observed by Zhang et al, (2019) in *Brassica rapa* L. for the visual color scores and color space values, as they were able to identify the same QTL regions for seed coat color. In contrast to our results, Sohi et al. (2022) identified one major and two minor QTLs for anthocyanin reflective index of guava leaves in LG3, LG6 and LG8 as the presence of anthocyanin is well known to render leaves with purple color (Shiva et al., 2017).

We also identified a major QTL cluster in LG2 that contained QTLs for visual pulp color (PC), PUL, PUA and PUB with the explained phenotypic variance at 43.2, 27.9, 35.4 and 17.2%, respectively. In addition, the Kruskal–Wallis statistic (K*) ranged between 49.7 and 61 (*P*<0.000005) for these pulp color attributes. These observations are strongly suggestive that these QTL regions might harbor candidate genes that are responsible for pulp color traits in guava. The pulp color QTLs were linked to SNP markers that were contributed from chromosomes 10 and 11 of guava genome. From our results, we can assume that the chromosome number and LG number that harbors the QTL regions for color-related traits may not have synteny. It was recently proposed that the segments/regions of the guava linkage map contained LGs that acquired segments from different chromosomes (Medeiros et al., 2022). Hence it would be interesting to further investigate the position of the identified QTL clusters in the guava genome and the candidate genes associated with color traits. Overall, the results of QTL analysis demonstrate that visual scoring of color is comparable with color reader-based values. However, color reader values help in fine dissection of various color parameters and identification of minor effect QTLs.

### 4.4 Candidate genes identification

Functional annotation of QTL associated genes identified TFs that act as regulator molecules in carotenoid and anthocyanin biosynthetic pathways. In our study, TFs like *bHLH*, *WD* repeats, *Myb*, *HD-ZIP*, *AP2/ERF*s, and *NAC* domain-containing proteins were found in the QTL regions of leaf, peel and pulp (**Table 6**). Flavanoids biosynthesis, in particular to anthocyanin is reported to be under the control of a MYB-bHLH-WDR transcription regulatory complex for the activation of structural genes involved in anthocyanin biosynthesis (Quattrocchio et al., 2006). Similarly, anthocyanin and carotenoid accumulation in fruits were reported to be under the regulatory control of myb genes in fruit crops like apple (Li et al., 2019), pear (Zhai et al., 2016), kiwi (Peng et al., 2019), peach (Zhao et al., 2020) etc. An essential enzyme in the shikimate pathway that generates homogentisate is 4-hydroxyphenylpyruvate dioxygenase (HPPD). Homogentisate is a precursor for plastoquinones (PQ) which are crucial electron acceptors required for carotenoid biosynthesis (Burke and Bell, 2014). The QTL regions were highly enriched in HPPD (**Supplementary Tables 3**, **4**), with phytoene synthase found in close proximity. Phytoene synthases (PSY) are key enzymes in carotenoid biosynthesis pathway that are under the positive transcriptional control of TFs like MYB, bHLH, NAC families and AP2/ERFs in apple (Ampomah-Dwamena et al., 2022; Dang et al., 2021). In addition, a pentatricopeptide-repeat region (PRR) was observed in peel QTL regions, which has been reported to be involved in fruit ripening and flesh color development in melons (Galpaz et al., 2018). Glycosyltransferases, the essential enzymes needed for anthocyanin formation from anthocyanidins (Liu et al., 2018) were also found in the QTL regions. Pathway enrichment and gene ontology analysis indicate that the QTLs identified are strongly linked to the coloration in leaf, peel and pulp of guava hybrids due to the presence of structural genes and TFs involved in production of pigments like chlorophylls, carotenoids and anthocyanins. The markers associated with these QTLs can serve as potential candidates for marker assisted breeding for colored genotypes in guava.

## 5 Conclusion

Advanced breeding techniques for guava necessitates the identification of markers associated to important fruit traits. Color of leaf, fruit peel and pulp are important horticultural traits for breeders and consumers as it renders an attractive appearance to the fruit and harbors enhanced nutritional values. This study has reported QTLs that are associated with leaf, fruit peel and pulp color traits. Further, it demonstrated that GBS approach can be effectively used for genetic linkage map construction and QTL mapping in guava. The QTLs identified for leaf and fruit color harbor genes like phytoene synthase, HPPD and TFs like *Myb, bHLH*, *WD* repeats, *HD-ZIP*, *AP2/ERF*s, and *NAC*s which are involved in lycopene and anthocyanin biosynthesis. Since, the biosynthesis and regulatory mechanisms in guava leaf and fruit colors are largely unknown, QTLs identified in this study can help in understanding molecular mechanism and develop marker-assisted selection for pulp color, in breeding programs for screening, and faster identification of desired progenies.

## Data availability statement

The datasets presented in this study can be found in online repositories. The names of the repository/repositories and accession number(s) can be found below: European Variation Archive (EVA) at EMBL-EBI under accession number PRJEB70471.

## Author contributions

MM: Data curation, Formal Analysis, Investigation, Methodology, Writing – original draft. DE: Formal Analysis, Software, Writing – review & editing. VC: Resources, Writing – review & editing, Conceptualization, Methodology. KS: Funding acquisition, Writing – review & editing, Methodology. DS: Methodology, Writing – review & editing. KR: Conceptualization, Funding acquisition, Supervision, Writing – review & editing, Methodology.

## References

[B1] Albert N. W. Lafferty D. J. Moss. S. M.A. Davies K. M. (2022). Flavonoids – flowers, fruit, forage and the future. J. R. Soc N. Z. 53, 304–331. doi: 10.1080/03036758.2022.2034654 PMC11459809 39439482

[B2] Ampomah-Dwamena C. Tomes S. Thrimawithana A. H. Elborough C. Bhargava N. Rebstock R.. (2022). Overexpression of PSY1 increases fruit skin and flesh carotenoid content and reveals associated transcription factors in apple (Malus × domestica). Front. Plant Sci. 13. doi: 10.3389/fpls.2022.967143 PMC9520574 36186009

[B3] Battistoni B. Salazar J. Vega W. Valderrama-Soto D. Jiménez-Muñoz P. Sepúlveda-González A.. (2022). An upgraded, highly saturated linkage map of Japanese plum (*Prunus salicina* lindl.), and identification of a new major locus controlling the flavan-3-ol composition in fruits. Front. Plant Sci. 13, 805744. doi: 10.3389/fpls.2022.805744 35310655 PMC8931734

[B4] Browning B. L. Zhou Y. Browning S. R. (2018). A one-penny imputed genome from next generation reference panels. Am. J. Hum. Genet. 103, 338–348. doi: 10.1016/j.ajhg.2018.07.015 30100085 PMC6128308

[B5] Burke I. C. Bell J. L. (2014). Plant health management: herbicides. In: van Alfen N. K. (Ed.) Encyclopedia Agric. Food Syst. (pp. 425–440). Academic Press.

[B6] Chen Z. Yu L. Liu W. Zhang J. Wang N. Chen X. (2021). Research progress of fruit color development in apple (*Malus domestica* Borkh.). Plant Physiol. Biochem. 162, 267–279. doi: 10.1016/j.plaphy.2021.02.033 33711720

[B7] Chen S. Zhou Y. Chen Y. Gu J. (2018). Fastp: an ultra-fast all-in-one FASTQ preprocessor. Bioinform 34, i884–i890. doi: 10.1093/bioinformatics/bty560 PMC6129281 30423086

[B8] Choo W. S. (2018). “Fruit pigment changes during ripening,” in Encyclopedia of food chemistry (Amsterdam Netherlands: Elsevier), 117–123.

[B9] Coser S. M. da Silva Ferreira M. F. Ferreira A. Mitre L. K. Carvalho C. R. Clarindo W. R. (2012). Assessment of genetic diversity in psidium guajava l. using different approaches. Scientia Hortic. 148, 223–229.

[B10] Dang Q. Sha H. Nie J. Wang Y. Yuan Y. Jia D. (2021). An apple (*Malus domestica*) AP2/ERF transcription factor modulates carotenoid accumulation. Hortic. Res. 8, 1–12. doi: 10.1038/s41438-021-00694-w 34611138 PMC8492665

[B11] Elshire R. J. Glaubitz J. C. Sun Q. Poland J. A. Kawamoto K. Buckler E. S.. (2011). A robust, simple genotyping-bysequencing (GBS) approach for high diversity species. PloS One 6, e19379. doi: 10.1371/journal.pone.0019379 21573248 PMC3087801

[B12] FAO (2023). Major Tropical Fruits Market Review – Preliminary results 2022 (Rome: FAO).

[B13] Feng C. Feng C. Lin X. Liu S. Li Y. Kang M. (2021). A chromosome-level genome assembly provides insights into ascorbic acid accumulation and fruit softening in guava (*Psidium guajava*). Plant Biotechnol. J. 19, 717–730. doi: 10.1111/pbi.13498 33098334 PMC8051600

[B14] Galpaz N. Gonda I. Shem-Tov D. Barad O. Tzuri G. Lev S.. (2018). Deciphering genetic factors that determine melon fruit-quality traits using RNA-Seq-based high-resolution QTL and eQTL mapping. Plant J. 94, 169–191. doi: 10.1111/tpj.13838 29385635

[B15] Ge S. X. Jung D. Yao R. (2020). ShinyGO: a graphical gene-set enrichment tool for animals and plants. Bioinformatics 36, 2628–2629. doi: 10.1093/bioinformatics/btz931 31882993 PMC7178415

[B16] Gonzali S. Perata P. (2021). Fruit colour and novel mechanisms of genetic regulation of pigment production in tomato fruits. Horticulturae 7, 259. doi: 10.3390/horticulturae7080259

[B17] Han H. Oh Y. Kim K. Oh S. Cho S. Kim Y. K.. (2019). Integrated genetic linkage maps for Korean pears (Pyrus hybrid) using GBS-based SNPs and SSRs. Hortic. Environ. Biotechnol. 60, 779–786. doi: 10.1007/s13580-019-00171-3

[B18] Kherwar D. Usha K. Mithra S. A. Singh B. (2018). Microsatellite (SSR) marker assisted assessment of population structure and genetic diversity for morpho-physiological traits in guava (Psidium guajava l.). J. Plant Biochem. Biotechnol. 27, 284–292.

[B19] Kumar C. Kumar R. Singh S. K. Goswami A. K. Nagaraja A. Paliwal R.. (2020). Development of novel g-SSR markers in guava (*Psidium guajava* L.) cv. Allahabad Safeda and their application in genetic diversity, population structure and cross species transferability studies. PloS One 15, e0237538. doi: 10.1371/journal.pone.0237538 32804981 PMC7431106

[B20] Kunihisa M. Takita Y. Yamaguchi N. Okada H. Sato M. Komori S.. (2019). The use of a fertile doubled haploid apple line for QTL analysis of fruit traits. Breed. Sci. 69, 410–419. doi: 10.1270/jsbbs.18197 31598073 PMC6776154

[B21] Labadie M. Vallin G. Potier A. Petit A. Ring L. Hoffmann T.. (2022). High resolution quantitative trait locus mapping and whole genome sequencing enable the design of an anthocyanidin reductase-specific homoeo-allelic marker for fruit colour improvement in octoploid strawberry (Fragaria × ananassa). Front. Plant Sci. 13. doi: 10.3389/fpls.2022.869655 PMC8972132 35371183

[B22] Li W. F. Ning G. X. Mao J. Guo Z. G. Zhou Q. Chen B. H. (2019). Whole-genome DNA methylation patterns and complex associations with gene expression associated with anthocyanin biosynthesis in apple fruit skin. Planta 250, 1833–1847. doi: 10.1007/s00425-019-03266-4 31471637

[B23] Liu Y. Tikunov Y. Schouten R. E. Marcelis L. F. Visser R. G. Bovy A. (2018). Anthocyanin biosynthesis and degradation mechanisms in Solanaceous vegetables: A review. Front. Chem. 6, 52. doi: 10.3389/fchem.2018.00052 29594099 PMC5855062

[B24] Luo X. Xu L. Wang Y. Dong J. Chen Y. Tang M.. (2020). An ultra-high-density genetic map provides insights into genome synteny, recombination landscape and taproot peel color in radish (*Raphanus sativus* L.). Plant Biotechnol. J. 18, 274–286. doi: 10.1111/pbi.13195 31218798 PMC6920339

[B25] Maan S. S. Brar J. S. Mittal A. Gill M. I. S. Arora N. K. Sohi H. S.. (2023). Construction of a genetic linkage map and QTL mapping of fruit quality traits in guava (*Psidium guajava* L.). Front. Plant Sci. 14, 1123274. doi: 10.3389/fpls.2023.1123274 37426984 PMC10324979

[B26] Mathiazhagan M. Chinnaiyan V. Ravishankar K. V. (2023). “Guava: A nutraceutical-rich underutilized fruit crop,” in Compendium of crop genome designing for nutraceuticals. Ed. Kole C. (Singapore: Springer), 1069–1096. doi: 10.1007/978-981-19-3627-2_42-1

[B27] Mathiazhagan M. Chidambara B. Hunashikatti L. R. Ravishankar K. V. (2021). Genomic approaches for improvement of tropical fruits: fruit quality, shelf life and nutrient content. Genes 12, 1881. doi: 10.3390/genes12121881 34946829 PMC8701245

[B28] Mathiazhagan M. Padala S. Doddahejjaji S. G. C. Murugan S. Makki D. R. Kundapura R. V. (2022). Omics of mango: A tropical fruit tree. in Omics in Horticultural Crops. Eds. Rout G. R. Peter K. V. (London: Academic Press), 427–448. doi: 10.1016/B978-0-323-89905-5.00013-6

[B29] Medeiros F. L. B. Santos C. A. F. Costa A. E. S. (2022). Comparing SNP-based genetic linkage and physical maps in guava (Psidium guajava). Genet. Mol. Res. 21 (2), gmr19033. doi: 10.4238/gmr19033

[B30] Nawaz R. Abbasi N. A. Khan M. R. Ali I. Hasan S. Z. U. Hayat A. (2020). Color development in ‘Feutrell’s Early’ (*Citrus reticulata* Blanco) affects peel composition and juice biochemical properties. Int. J. Fruit Sci. 20, 871–890. doi: 10.1080/15538362.2019.1699490

[B31] Ntladi S. M. Human J. P. Bester C. Vervalle J. Roodt-Wilding R. Tobutt K. R. (2018). Quantitative trait loci (QTL) mapping of blush peel and flowering time in a European pear (*Pyrus communis*) progeny of ‘Flamingo’×’Abate Fetel’. Tree Genet. Genomes. 14, 1–24. doi: 10.1007/s11295-018-1280-y

[B32] Oh S. Ahn S. Han H. Kim K. Kim S. A. Kim D. (2023). Genetic linkage maps and QTLs associated with fruit peel color and acidity in apple (Malus × domestica). Hortic. Environ. Biotechnol. 64, 299–310. doi: 10.1007/s13580-022-00473-z

[B33] Olivoto T. Lúcio A. D. C. (2020). Metan: An R package for multi-environment trial analysis. Methods Ecol. Evol. 11, 783–789. doi: 10.1111/2041-210X.13384

[B34] Padmakar B. Kanupriya C. Latha P. M. Prashant K. S. Dinesh M. R. Sailaja D.. (2015). Development of SRAP and SSR marker-based genetic linkage maps of guava (Psidium guajava l.). Scientia Hortic. 192, 158–165.

[B35] Padmakar B. Kanupriya C. Latha P. M. Vasugi C. Dinesh M. R. Sailaja D.. (2016). Enrichment of genetic linkage maps and mapping QTLs specific to seed strength-hardness/softness-in guava (*Psidium guajava L.*). J. Hortic. Sci. 11, 13–20. doi: 10.24154/jhs.v11i1.96

[B36] Peng Y. Lin-Wang K. Cooney J. M. Wang T. Espley R. V. Allan A. C. (2019). Differential regulation of the anthocyanin profile in purple kiwifruit (Actinidia species). Horti. Res. 6, 3. doi: 10.1038/s41438-018-0076-4 PMC6312553 30622721

[B37] Popat R. Patel R. Parmar D. (2020). Variability: Genetic Variability Analysis for Plant Breeding Research, R package version 0.1.0. Available at: http://cran.r-project.org/package=variability.

[B38] Quattrocchio F. Baudry A. Lepiniec L. Grotewold E. (2006). “The regulation of flavonoid biosynthesis,” in The Science of Flavonoids. Ed. Grotewold E. (Springer, New York, NY). doi: 10.1007/978-0-387-28822-2_4

[B39] Rajan S. Yadava L. P. Kumar R. Saxena S. K. (2005). Selection possibilities for seed content-a determinant of fresh fruit quality in guava (*Psidium guajava* L.). J. Appl. Hortic. 7, 52–54. doi: 10.37855/jah.2005.v07i01.14

[B40] Ran S. Sharma J. R. Jakhar M. S. (2017). Assessment of genetic diversity and diversity relationship in different varieties of guava using morphological characterization. Plant Arch. 17, 307–311.

[B41] Ravishankar K. V. Anand L. Dinesh M. R. (2000). Assessment of genetic relatedness among mango cultivars of India using RAPD markers. J. Hortic. Sci. Biotechnol. 75, 198–201. doi: 10.1080/14620316.2000.11511223

[B42] Ritter E. Herran A. Valdés-Infante J. Rodríguez-Medina M. N. Briceño A. Fermin G.. (2010). Comparative linkage mapping in three guava mapping populations and construction of an integrated reference map in guava. Acta Hortic 849, 175–182.

[B43] Rochette N. C. Rivera-Colón A. G. Catchen J. M. (2019). Stacks 2: Analytical methods for paired-end sequencing improve RADseq-based population genomics. Mol. Ecol. 28, 4737–4754. doi: 10.1111/mec.15253 31550391

[B44] Rodriguez N. Valdes-Infante J. Becker D. Velazquez B. Gonzalez G. Sourd D.. (2007). Characterization of guava accessions by SSR markers, extension of the molecular linkage map, and mapping of QTLs for vegetative and reproductive characters. Acta. Hortic. 735, 201–216.

[B45] Rojas-Garbanzo C. Rodríguez L. Pérez A. M. Mayorga-Gross A. L. Vásquez-Chaves V. Fuentes E.. (2021). Anti-platelet activity and chemical characterization by UPLC-DAD-ESI-QTOF-MS of the main polyphenols in extracts from psidium leaves and fruits. Food Res. Int. 141, 110070. 33641960 10.1016/j.foodres.2020.110070

[B46] Sampangi-Ramaiah M. H. Shivashankara K. S. Rekha A. Laxman R. H. Dayanandhi E. Ravishankar K. V. (2023). High-density GBS-based genetic linkage map construction and QTL identification associated with leaf cuticular wax, adaxial stomatal density and leaf water retention capacity in banana. Sci. Hortic. 321, 112205. doi: 10.1016/j.scienta.2023.112205

[B47] Shiva B. Nagaraja A. Srivastav M. Kumari S. Goswami A. K. Singh R.. (2017). Characterization of guava (*Psidium guajava*) germplasm based on leaf and fruit parameters. Ind. J. Agric. Sci. 87, 634–638. doi: 10.56093/ijas.v87i5.70172

[B48] Sohi H. S. Gill M. I. S. Chhuneja P. Arora N. K. Maan S. S. Singh J. (2022). Construction of genetic linkage map and mapping QTL specific to leaf anthocyanin colouration in mapping population ‘Allahabad Safeda’×‘Purple guava (Local)’of guava (Psidium guajava l.). Plants 11 (15), 2014. 35956491 10.3390/plants11152014 PMC9370526

[B49] Srivastav M. Radadiya N. Ramachandra S. Jayaswal P. K. Singh N. Singh S.. (2023). High resolution mapping of QTLs for fruit color and firmness in Amrapali/Sensation mango hybrids. Front. Plant Sci. 14, 1135285. doi: 10.3389/fpls.2023.1135285 37351213 PMC10282835

[B50] Subramanyam M. D. Iyer C. P. A. (1992). Studies on inheritance in guava (*Psidium quajava* L.). Fruit Breed. Genet. 317, 255–258.

[B51] Tello D. Gil J. Loaiza C. D. Riascos J. J. Cardozo N. Duitama J. (2019). NGSEP3: accurate variant calling across species and sequencing protocols. Bioinform 35, 4716–4723. doi: 10.1093/bioinformatics/btz275 PMC6853766 31099384

[B52] Thakre M. MK R. Senapati R. Rudra S. G. Saha S. Nagaraja A.. (2023). Pigment composition analysis of fruit pulp in the recombinant progenies reveals the polygenic nature of pulp color inheritance in guava (*Psidium guajava* L.). Tree Genet. Genome. 19, 20. doi: 10.1007/s11295-023-01595-w

[B53] Toppo P. Xaxa S. Singh A. Singh D. Prasad V. M. (2018). Studies on value added products of guava cheese. Int. J. Pure App. Biosci. 6, 471–474.

[B54] Underhill A. N. Hirsch C. D. Clark M. D. (2020). Evaluating and mapping grape color using image-based phenotyping. Plant Phenom. 2020. doi: 10.34133/2020/8086309 PMC7706331 33313563

[B55] Valdés-Infante J. Sourd D. Rodriguez J. Becker D. Rohde W. Ritter E. (2003). Molecular characterization of Cuban accessions of guava (*Psidium guajava L.*), establishment of a first molecular linkage map and mapping of QTLs for vegetative characters. J. Genet. Breed 57, 349–357.

[B56] Van Ooijen J. W. (2009). Map QTL 6: software for the mapping of quantitative trait loci in experimental populations of diploid species (Netherlands: Wageningen).

[B57] Van Ooijen J. W. (2011). Multipoint maximum likelihood mapping in a full-sib family of an outbreeding species. Genet. Res. 93, 343–349. doi: 10.1017/S0016672311000279 21878144

[B58] Zhai R. Wang Z. Zhang S. Meng G. Song L. Wang Z.. (2016). Two MYB transcription factors regulate flavonoid biosynthesis in pear fruit (*Pyrus bretschneideri* Rehd.). J. Exp. Botany. 67, 1275–1284. doi: 10.1093/jxb/erv524 26687179

[B59] Zhang Y. Sun Y. Sun J. Feng H. Wang Y. (2019). Identification and validation of major and minor QTLs controlling seed coat color in brassica rapa l. Breed. Sci. 69 (1), 47–54. 31086483 10.1270/jsbbs.18108 PMC6507729

[B60] Zhao Y. Dong W. Zhu Y. Allan A. C. Lin-Wang K. Xu C. (2020). PpGST1, an anthocyanin-related glutathione S-transferase gene, is essential for fruit coloration in peach. Plant Biotechnol. J. 18, 1284–1295. doi: 10.1111/pbi.13291 31693790 PMC7152611

[B61] Zhao X. Zhang Y. Long T. Wang S. Yang J. (2022). Regulation mechanism of plant pigments biosynthesis: anthocyanins, carotenoids, and betalains. Metabolites 12, 871. doi: 10.3390/metabo12090871 36144275 PMC9506007

[B62] Zhu X. Weng Q. Bush D. Zhou C. Zhao H. Wang P.. (2023). High-density genetic linkage mapping reveals low stability of QTLs across environments for economic traits in Eucalyptus. Front. Plant Sci. 13, 1099705. doi: 10.3389/fpls.2022.1099705 37082511 PMC10112524

