# Supplementary figures and images for: A high-density linkage map construction in guava (Psidium guajava L.) using genotyping by sequencing and identification of QTLs for leaf, peel, and pulp color in an intervarietal mapping population
Source: Front Plant Sci. 2024 Feb 27;15:1335715. doi: 10.3389/fpls.2024.1335715 (PMC10927721; doi:10.3389/fpls.2024.1335715)

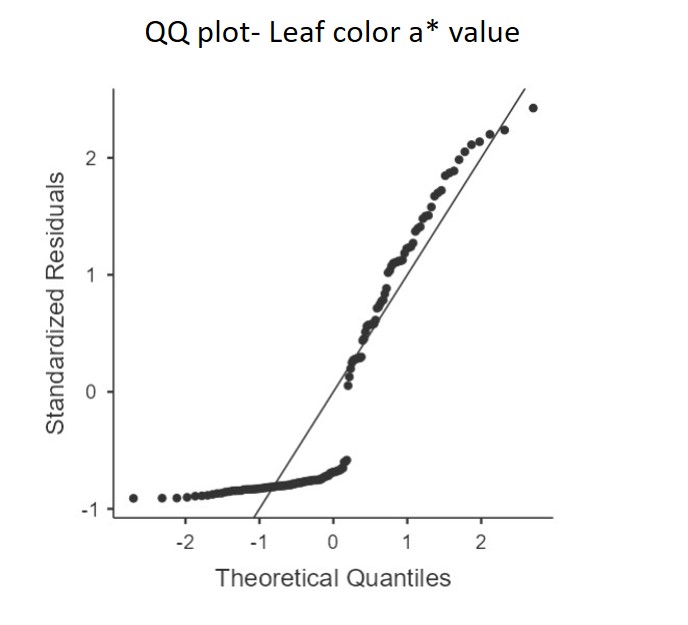

Supplement: Supplementary file 1 [file Image_1.jpeg]

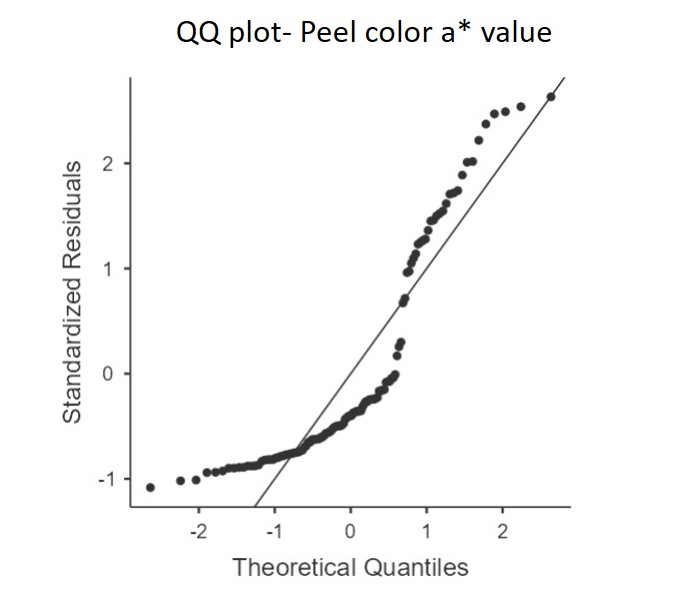

Supplement: Supplementary file 2 [file Image_2.jpeg]

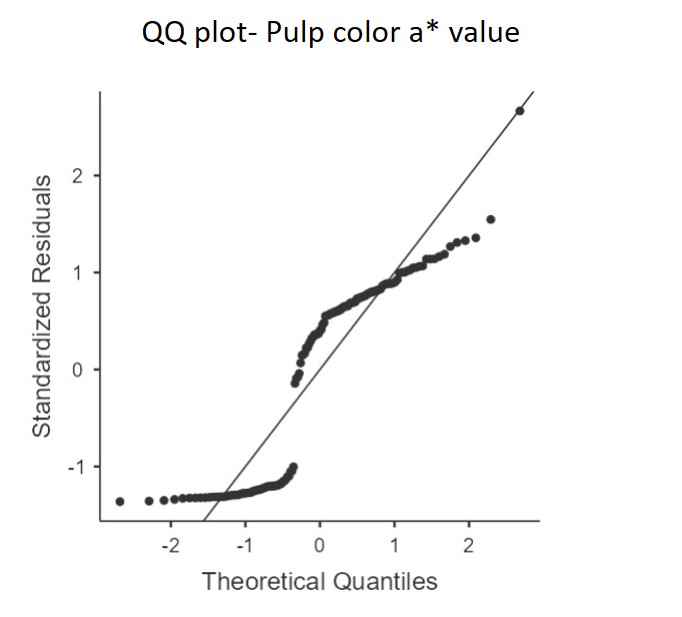

Supplement: Supplementary file 3 [file Image_3.jpeg]

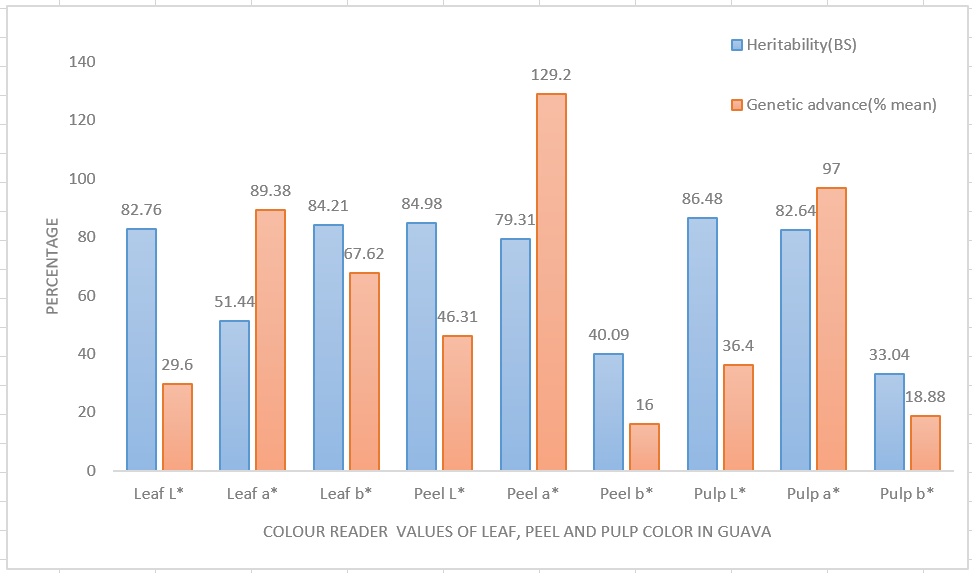

Supplement: Supplementary file 4 [file Image_4.png]

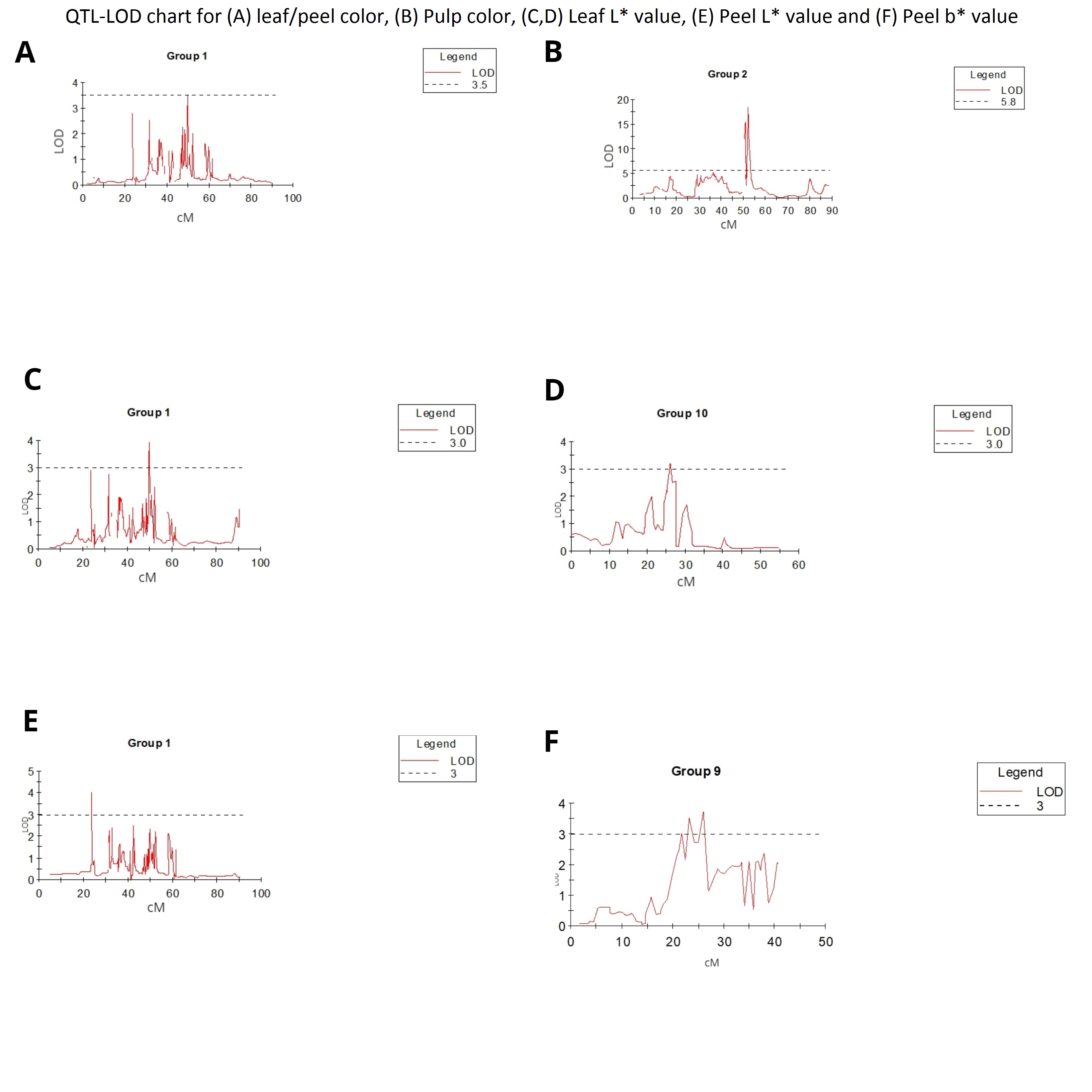

Supplement: Supplementary file 5 [file Image_5.jpg]

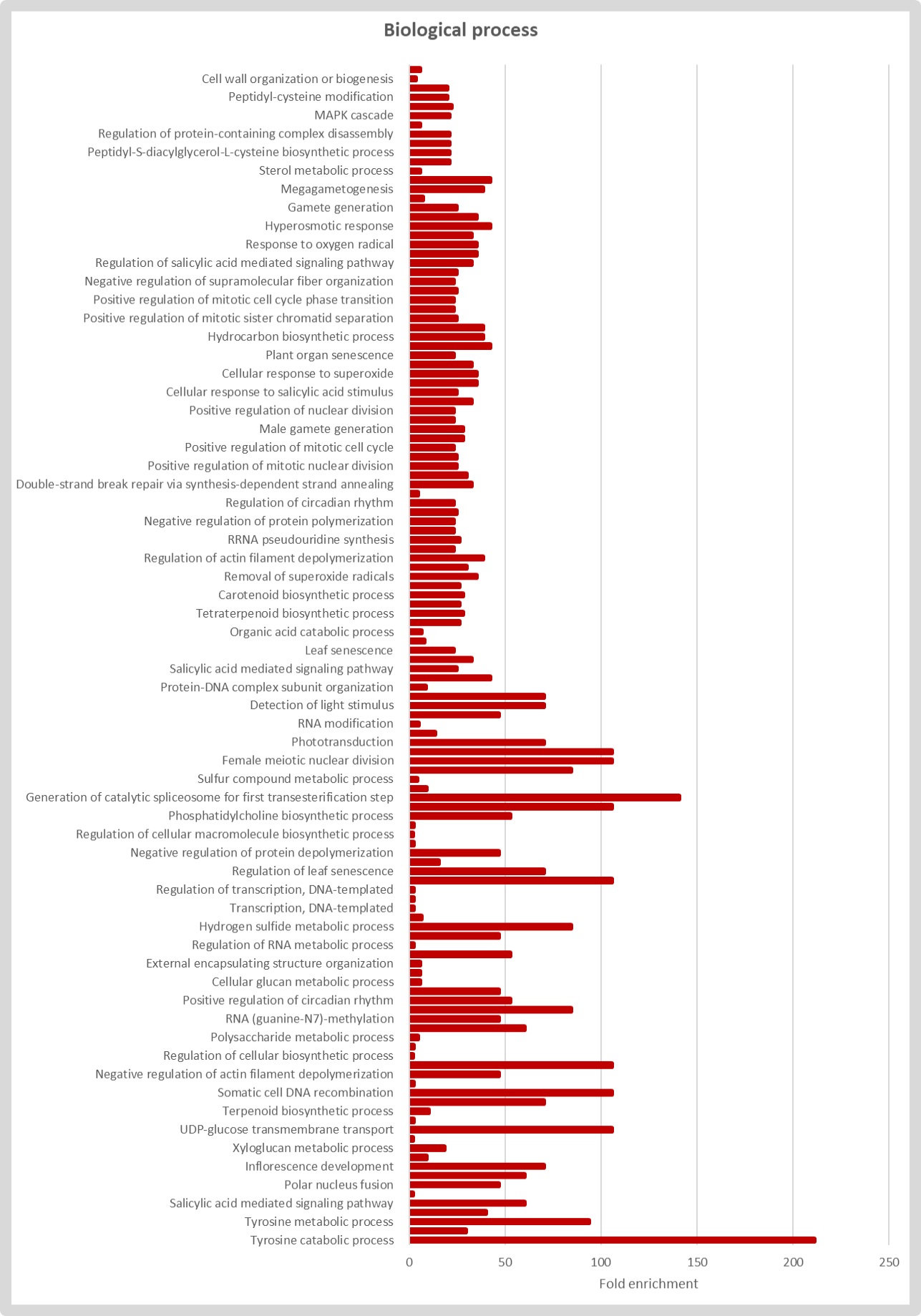

Supplement: Supplementary file 6 [file Image_6.jpeg]

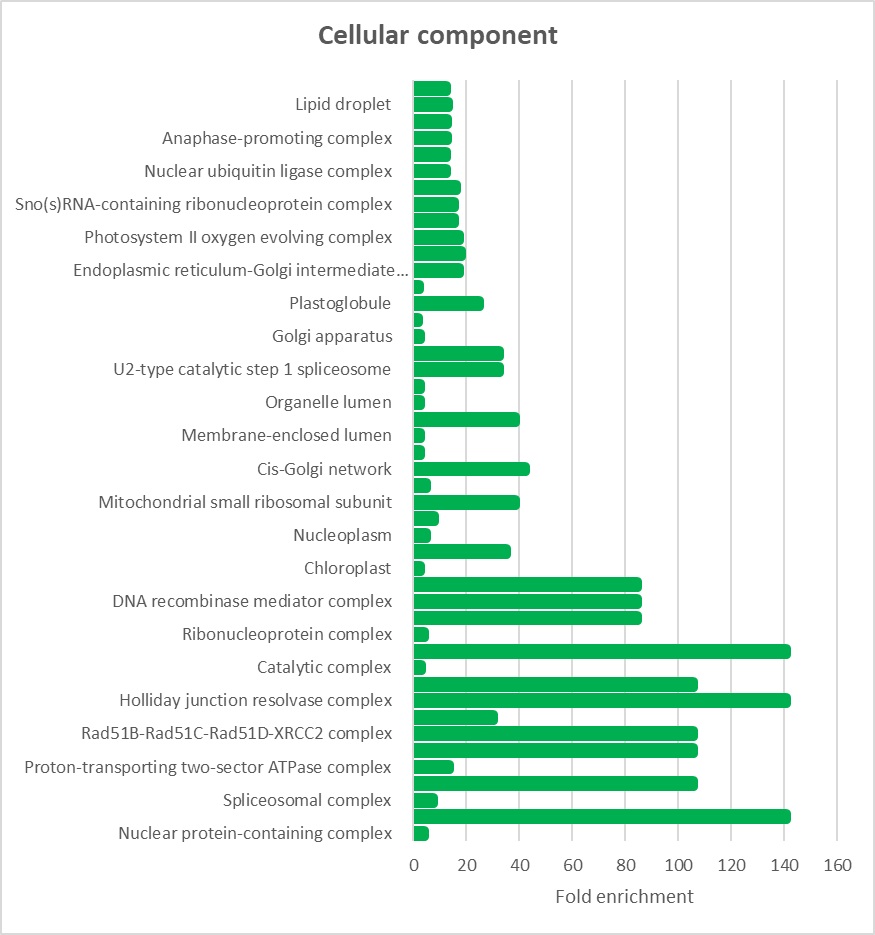

Supplement: Supplementary file 7 [file Image_7.jpeg]

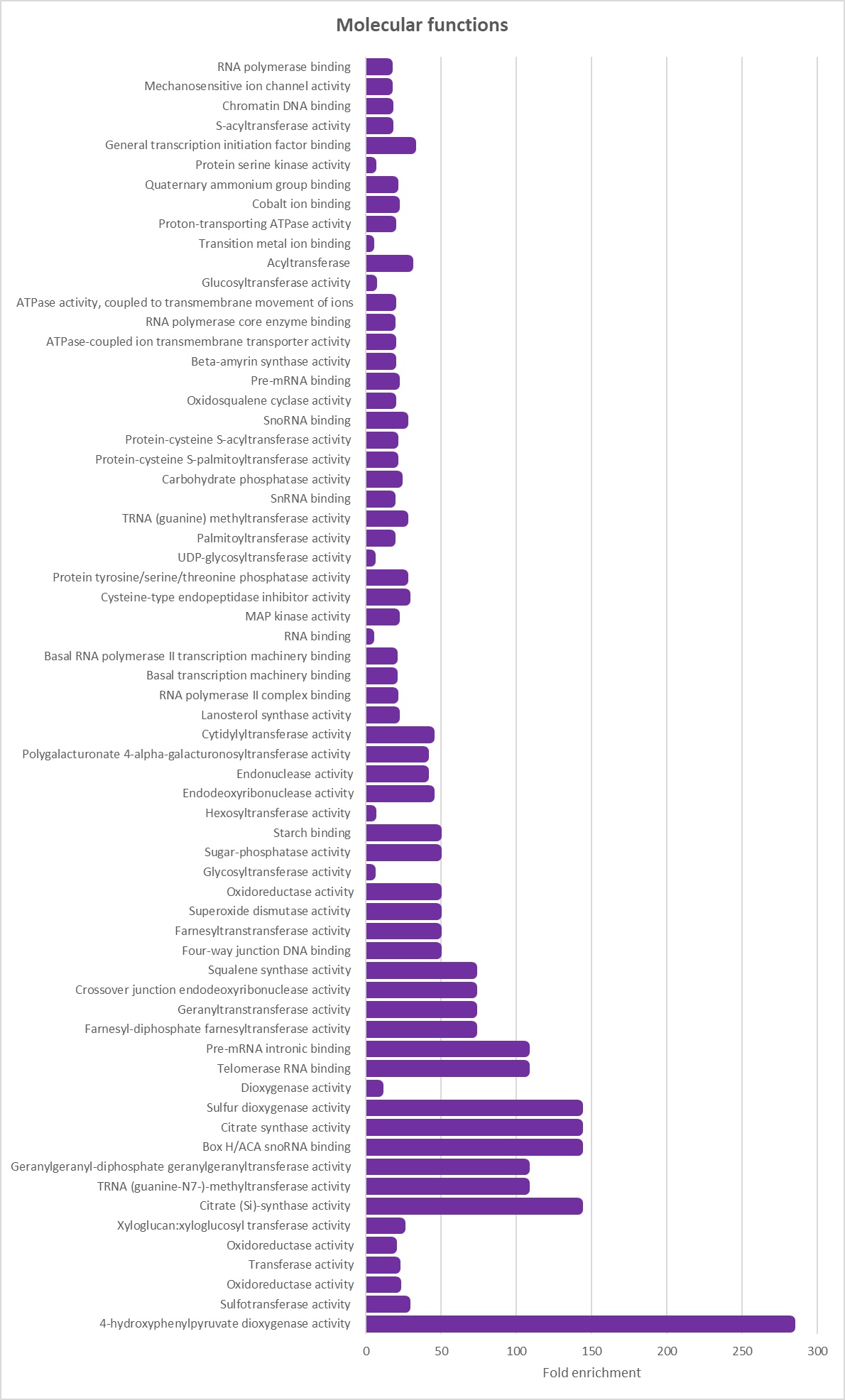

Supplement: Supplementary file 8 [file Image_8.jpeg]
